# Comprehensive Cultivation of the Swine Gut Microbiome Reveals High Bacterial Diversity and Guides Bacterial Isolation in Pigs

**DOI:** 10.1128/mSystems.00477-21

**Published:** 2021-07-20

**Authors:** Xiaofan Wang, Samantha Howe, Xiaoyuan Wei, Feilong Deng, Tsungcheng Tsai, Jianmin Chai, Yingping Xiao, Hua Yang, Charles V. Maxwell, Ying Li, Jiangchao Zhao

**Affiliations:** a Department of Animal Science, Division of Agriculture, University of Arkansas, Fayetteville, Arkansas, USA; b Guangdong Provincial Key Laboratory of Animal Molecular Design and Precise Breeding, College of Life Science and Engineering, Foshan University, Foshan, China; c State Key Laboratory for Managing Biotic and Chemical Threats to the Quality and Safety of Agro-products, Institute of Agro-product Safety and Nutrition, Zhejiang Academy of Agricultural Sciences, Hangzhou, China; University of Pittsburgh Medical Center

**Keywords:** swine gut microbiome, diversity, culturability, culturomics, novel strain

## Abstract

Despite the substantial progress made in human gut culturomics, little is known about the culturability of the swine gut microbiota. In this study, we cultured swine gut microbiota using 53 bacterial cultivation methods with different medium and gas combinations from three pigs at four different growth stages. Both culture-dependent (CD; colony mixtures from each method) and culture-independent (CI; original fecal suspensions) samples were subjected to 16S rRNA gene amplicon sequencing. Increasing microbial diversities were observed in both CI and CD samples from successive growth stages. While a total of 378, 482, 565, and 555 bacterial amplicon sequence variants (ASVs) were observed in the CI samples, higher microbial diversities (415, 675, 808, and 823 observed ASVs) were detected using the CD methods at the lactation, nursery, growing, and finishing stages, respectively. We constructed reference culture maps showing the preferred cultivation conditions for specific bacterial taxa and examined the effects of culturing factors such as oxygen, medium, donor pig age, antibiotics, and blood culture preincubation on swine gut microbiota cultivation. We focused on a wide range of beneficial bacteria, chose 1,299 colonies based on the reference map, and Sanger sequenced their 16S rRNA genes. These isolates clustered into 148 different bacterial taxa covering 28 genera. We observed 11, 19, 33, and 25 pairs of cooccurring ASVs in both CD and CI samples at four successive growth stages. This study provides guidance in culturing the swine gut microbiota of interest, which is critical when characterizing their functions in this important animal species.

**IMPORTANCE** The swine gut microbiome has been the focus of many investigations due to the fact that pigs serve as both an excellent biomedical model for human diseases and an important protein source. Substantial progress has been made in swine gut microbiome studies using next-generation sequencing-based culture-independent approaches, but little is known about the culturability of the swine gut microbiota. To understand their roles in swine production, it is critical to culture bacterial strains of interest. In this study, we cultured the gut microbiota from pigs at different growth stages using 53 bacterial cultivation methods with different medium and gas combinations. This study provides evidence that the swine gut microbiota is much more diverse based on a culture-dependent approach than previously known. It provides preliminary guidance for isolating certain bacteria of interest from pigs, which is critical in establishing causal relationships between the gut microbiota and the health status of pigs.

## INTRODUCTION

The advent of next- and third-generation sequencing techniques has dramatically improved our understanding of the important roles that the gut microbiome plays in human and animal health and diseases (1–6). To prove the beneficial or detrimental roles of specific bacteria, it is important to isolate these bacteria to perform *in vitro* and *in vivo* validations. Conventional culture-based studies have isolated only a small portion of bacteria, making it difficult to investigate complex community functions (7, 8). Therefore, despite the tremendous progress in identifying bacterial associations with host phenotypes such as health status and growth performance uncovered by culture-independent (CI) analyses, much still remains unknown regarding the functions of these bacteria; thus, validation through pure cultures is imperative (9, 10). In addition, sequencing of pure bacterial cultures can also expand the reference genome bank for metagenomics data analysis (11, 12). Zou et al. (2019) isolated and sequenced over 6,000 human bacterial isolates using 11 different media under anaerobic conditions, which resulted in 1,520 reference genomes and remarkably increased the mapping rates of metagenomics reads (12).

Culturomics is a comprehensive approach that applies multiple culture conditions to grow bacterial colonies on different plates followed by their identification using matrix-assisted laser desorption ionization–time of flight (MALDI-TOF) and/or 16S rRNA gene sequencing (13–15). Remarkable progress in human gut microbiota culturomics has been achieved recently (9). Lau and colleagues developed 33 different media under both anaerobic and aerobic conditions, which together cultured over 95% of the human fecal bacteria with a relative abundance of over 0.1% (16). In another study, 10 cultivation methods using commercial basal media such as M9, Gut Microbiota medium (GMM), and Gifu anaerobic medium with different nutrient supplements recovered 88% of the human gut microbiota at the family level (17). Recently, Browne and colleagues (18) developed a novel workflow based on targeted phenotyping and large-scale whole-genome sequencing and revealed a substantial portion of culturable human gut microbiota. Using yeast extract, Casitone, and fatty acid (YCFA)-based medium, they isolated a total of 137 bacterial taxa, among which 45 are candidate novel species, and 90 species belong to the Human Microbiome Project’s “most wanted” bacteria (18). Ito et al. (19) successfully cultured eight bacterial species previously considered “uncultivable” by using commercial media such as De Man, Rogosa, and Sharpe agar (MRS), Gut Microbiota medium, and Columbia nalidixic acid agar (CNA). They found that 61% of the amplicon sequence variants (ASVs) in fecal samples can be recovered using 26 cultivation methods (19). Several recent culturing strategies have been developed for human gut microbiota cultivation, including a wide range of conditioned culture medium systems (14, 16, 17), an *in vitro* native gut habitat model (20), an isolation chip (21), a single-colony coculture technique (22), refreshed blood culture bottles (23), and various sample-collecting origins (14).

Since pigs serve as an important protein source for the human diet and as a biomedical model for human diseases, this has recently led to more extensive investigations of swine microbiota, improving our understanding of the ecology and functions of the gut microbiome in health and production (5, 24–26). Similar to the human gut microbiome, most swine gut microbiome studies are descriptive and correlation based. Although some fecal microbial transplantation (FMT)-based research has demonstrated the “causality” of the swine gut microbiome regarding several production traits (27), the exact bacterial taxa that play such roles remain unknown. Unlike human gut microbiome research, efforts to culture swine gut microbiota have been weak. It is unknown how many swine gut microbial species can be cultured *in vitro*. Specifically, how many of the abundant bacterial taxa in pigs can be cultured? Can we culture potentially beneficial bacteria that correlate with swine health and production for potential use as probiotics? Are there any rare bacteria that are often missed in culture-independent approaches? Can any novel bacterial species be unveiled by conventional culture conditions? Finally, can an *in vitro* culturing system reveal highly cooccurring microbes that have been discovered in live pigs, thus presenting a more flexible testing model in deciphering microbiota networks in the future?

To address these questions and to provide guidance for future swine gut microbiota cultivation, we tested 53 different combinations of culture media and environmental conditions (Table 1) and examined which bacteria could be potentially cultivated from pigs at four different growth stages. This study provides a foundation for future research aiming to understand the functions of specific bacterial strains and to establish causal relationships between these strains and swine gut health.

**TABLE 1:** List of 53 culture methods including media, additional supplements, and oxygen condition used for swine gut microbiome culture^*a*^

Medium full name	Abbreviation(s)	Additional supplement(s)	Aerobic	Anaerobic
Brain heart infusion agar	BHI1	No supplement	No. 1	No. 2
	BHI3	0.5 g/liter l-cysteine hydrochloride hydrate, 10 mg/liter hemin, and 1 mg/liter vitamin K	No. 3	No. 4
	BHI2	10 mg/liter colistin sulfate and 5 mg/liter nalidixic acid	No. 5	No. 6
	BHI4	0.5 g/liter l-cysteine hydrochloride hydrate, 10 mg/liter hemin, 1 mg/liter vitamin K, 10 mg/liter colistin sulfate, and 5 mg/liter nalidixic acid	No. 7	No. 8
0.2× diluted BHI	B2I	1 g/liter inulin	No. 9	No. 10
	B2P	0.5 g/liter pectin	No. 11	No. 12
	B2C	0.5 g/liter cellulose	No. 13	No. 14
	B2M	0.5 g/liter mucin	No. 15	No. 16
	B2S	0.5 g/liter starch	No. 17	No. 18
Columbia blood agar	CBA	5% sheep’s blood	No. 19	No. 20
Chocolate agar	CHOC	Hemoglobin 2%; supplement VX	No. 21	No. 22
Tryptic soy agar	TSY	0.5 g/liter l-cysteine hydrochloride hydrate, 10 mg/liter hemin, 1 mg/liter vitamin K, and 5 g/liter yeast extract	No. 23	No. 24
Fastidious anaerobe agar	FAA		No. 25	No. 26
Cooked meat agar	BEEF		No. 27	No. 28
*Bifidobacterium* selective medium	BSM			No. 29
Phenyletdyl alcohol agar	PEA	5% sheep’s blood	No. 30	No. 31
*Actinomyces* isolation agar	AIA		No. 32	No. 33
Colistin-nalidixic acid agar	CNA	5% sheep’s blood	No. 34	No. 35
McKay agar	MK			No. 36
de Man-Rogosa-Sharpe agar	MRS		No. 37	No. 38
*Bacteroides* bile esculin agar	BBE		No. 39	No. 40
Deoxycholate agar	DOC		No. 41	No. 42
Kanamycin-vancomycin laked blood agar	KVLB			No. 43
Modified Gifu-anaerobic agar	MGAM		No. 44	No. 45
Diluted MGAM	DMG	10× diluted MGAM	No. 46	No. 47
*Blautia* selection medium	BLAU	MGAM + 64 μg/ml sulfamethoxazole and 16 μg/ml ciprofloxacin	No. 48	No. 49
Blood bottle-preculture + MGAM	BBMGAM			No. 50
Blood bottle-preculture + CBA	BBCBA			No. 51
Blood bottle-preculture + DMG	BBDMG			No. 52
Blood bottle-preculture + BLAU	BBBLAUT^*b*^^,^^*c*^			No. 53

a Methods using agars including BSM, MK, KVLB, and those preincubated in blood culture bottle (i.e., BBBLAUT, BBCBA, BBDMG, and BBMGAM) were not used under aerobic condition due to growth of rare colonies. Culture methods 1 to 43 were selected from a previous publication (16); culture methods 44 to 49 were selected from reference 17; culture methods 50 to 53 were adopted from the work of Lagier et al. (14).

b BBBLAUT applied to only growing and finishing stages’ fecal microbial culture.

c The blood culture bottle was a Bactec Standard Anaerobic/F culture vial (BD, Sparks, MD, USA) supplemented with 4 ml of sterile sheep blood. After microbial solution injection, the anaerobic blood culture bottle was incubated under 37°C for 4 days. Then, a volume of 50 μl of cell mixture from blood culture bottles was used for further regular anaerobic culturing on BLAU agar for 3 days.

## RESULTS

### CD swine gut microbiota diversity.

We sequenced the pooled colonies from each plate, yielding a total of 11,788,125 high-quality reads with an average of 18,390 reads per sample. We then assigned these sequences to 1,850 ASVs with single-nucleotide resolution using the Deblur algorithm. After rarefaction, a total of 1,832 ASVs (2,884,500 total reads) from 641 samples, including 12 fecal samples at four growth stages and 629 culture-dependent (CD) samples (one CD sample using a finishing stage fecal inoculum under anaerobic condition on brain heart infusion 1 (BHI1) was removed from the 630 CD samples due to low sequencing depth) generated under different culture conditions, from these fecal samples, were kept for downstream analysis.

### (i) Total bacterial ASVs revealed by cultivation in pigs at each growth stage.

**FIG 1:**
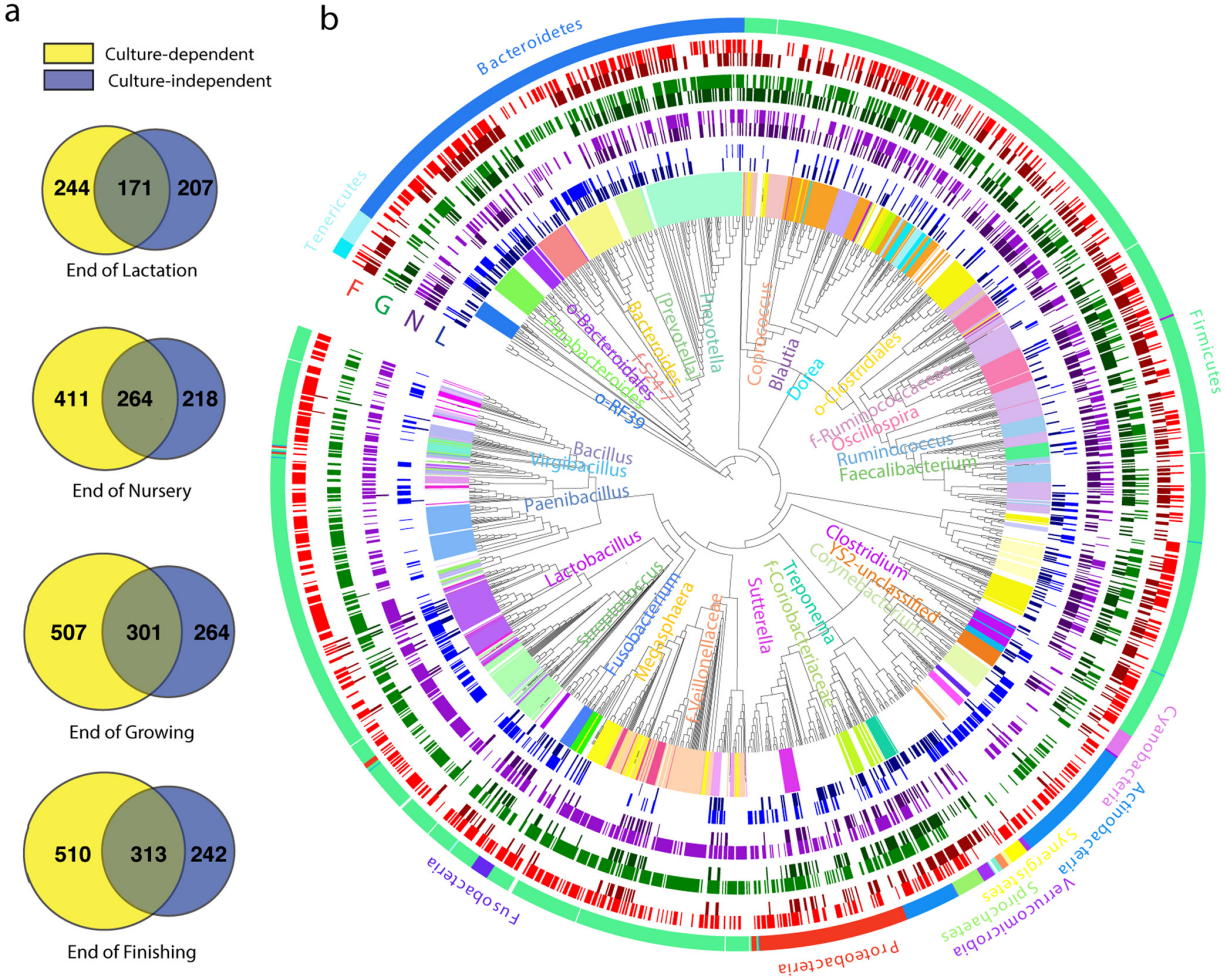
Culture-independent (CI) and culture-dependent (CD) view of the swine gut microbiome at four growth stages. Venn diagrams (a) show the number of detected bacterial ASVs with CI (blue) and CD (yellow) methods at each time point. Phylogenetic tree (b) shows bacterial ASVs detected by CI (dark color) and CD (light color) methods at the end of lactation (L; blue), nursery (N; purple), growing (G; green), and finishing (F; red) stages. The outermost ring is colored by phylum, while the innermost ring and labels are selectively colored by genus.

Consistent with a previous study (5), an increasing trend in gut microbiota richness was observed at the end of lactation, nursery, growing, and finishing stages with 378, 482, 565, and 555 bacterial ASVs, respectively, in feces (CI; Fig. 1a). Accordingly, the number of bacterial ASVs detected by CD also increased, with a total of 415, 675, 808, and 823 ASVs at four successive growth stages. Among these ASVs, 171, 264, 301, and 313 were shared by both CD and CI methods at these stages (Fig. 1a). CD methods, together with CI, remarkably increased the recovery of microbial richness in pigs, yielding a total of 622, 893, 1,072, and 1,065 bacterial ASVs at the four growth stages, respectively (Fig. 1b). Although interanimal variation at each stage was observed, overall, similar recovery patterns were observed consistently in all three individual pigs at all four time points (see Fig. S1a in the supplemental material). A steady increase in Shannon diversity index values was observed along with age by the CI and CD methods (Fig. S1b) while different patterns were found in Simpson evenness index values between these two methods (Fig. S1c). On average, 45.2, 54.8, 53.3, and 56.4% of CI-detected ASVs were observed on the plates at the end of lactation (day 20 [d20]), nursery (d61), growing (d116), and finishing (d174), respectively, using the 53 culturing methods. About 44 to 54% of total ASVs detected with CI methods at each time point still remain to be cultured (Fig. 1a and b) using our culturing methods. Of note, although many bacterial ASVs detected by CI methods are yet to be cultured, the CD technique did reveal more bacterial ASVs than CI (Fig. 1b and Fig. S2a). A similar pattern was also observed consistently at other phylogenetic levels, including order, family, and genus (Table 2). These groups of CD-only bacterial ASVs were widespread throughout most phyla and were associated with well-known genera such as *Bacillus*, *Lactobacillus*, and *Megasphaera* (Fig. 1b and Fig. S2a).

**TABLE 2:** Number of bacterial taxa at different taxonomic levels observed in swine gut using culture-dependent (CD) and -independent (CI) methods and recovery rates at each time point

Taxonomy, total observed	End of lactation, d20			End of nursery, d61			End of growing, d116			End of finishing, d174		
	CI	CD	Recovery rate, cultivable CI	CI	CD	Recovery rate, cultivable CI	CI	CD	Recovery rate, cultivable CI	CI	CD	Recovery rate, cultivable CI
Phylum, 16	16	11	71%	12	12	85%	14	8	47%	14	12	73%
Class, 26	23	17	74%	20	19	85%	22	15	55%	21	20	81%
Order, 42	30	22	67%	23	46	83%	28	40	54%	29	52	76%
Family, 90	55	59	80%	40	58	83%	46	59	65%	50	66	74%
Genus, 216	102	119	73%	86	116	81%	94	128	74%	96	151	77%

10.1128/mSystems.00477-21.1 FIG S1 Venn diagram charts (a) showing numbers of observed bacterial ASVs detected by culture-dependent (CD) and -independent (CI) methods for three pigs at four time points. Bar charts showing Shannon (b) and Simpson evenness (c) of CI and CD gut microbiota (all culture methods merged; CD) in three pigs at each time point (L, lactation; N, nursery; G, growing; F, finishing). Error bars presented the standard deviation between three replicates. Download FIG S1, PDF file, 2.4 MB. Copyright © 2021 Wang et al. 2021 Wang et al. https://creativecommons.org/licenses/by/4.0/ This content is distributed under the terms of the Creative Commons Attribution 4.0 International license.

10.1128/mSystems.00477-21.2 FIG S2 Culture-dependent methods revealed new ASVs not detected by culture-independent methods at each stage, shown as blue (lactation), purple (nursery), green (growing), and red (finishing) bands (a). The outermost ring is colored by phylum, while the innermost ring and labels (partially shown) are selectively colored by genus. Longitudinal dynamics of ASV56 *Bacteroides* detected by CI method (b) in swine gut (5) and both CI and CD methods (BBMGAM and pooled other methods; blue, anaerobic condition; red, aerobic condition; green, CI) in this study (c). Venn diagram (d) showing overlaps among three groups of core members (detected by CI or CD method in the current and previous study [5]). Download FIG S2, TIF file, 2.3 MB. Copyright © 2021 Wang et al. 2021 Wang et al. https://creativecommons.org/licenses/by/4.0/ This content is distributed under the terms of the Creative Commons Attribution 4.0 International license.

### (ii) Swine gut microbial composition revealed by culture-dependent and -independent approaches.

**FIG 2:**
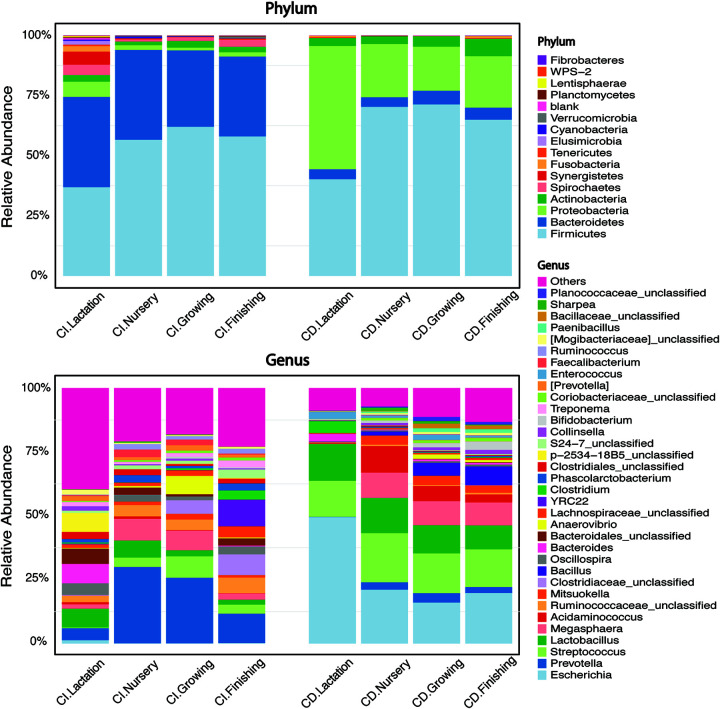
Stacked bar charts showing phyla (top panel) and top 20 genera (bottom panel) in swine feces collected at four time points by 16S rRNA gene amplicon sequencing using either culture-independent (CI) or -dependent (CD) methods. The CD method-generated stacked bar chart is an outcome of merging taxon from all 53 culture conditions. Bar height in each color represents the relative abundance of a bacterial taxon.

We detected a total of 16 phyla in the swine gut using CI methods (Fig. 2; Table 2). *Firmicutes* and *Bacteroidetes* (at 53 and 35%, respectively, averaged at four time points) were the two most dominant phyla detected by CI methods, whereas *Firmicutes* and *Proteobacteria* (averaged at 62 and 28%, respectively) were the top two abundant phyla revealed by CD methods, followed by *Bacteroidetes* and *Actinobacteria* (both at 5%). *Fusobacteria*, which ranked the fifth phylum using the CD method, was enriched to 0.8% using finishing stage feces, although it was not detected in the original fecal suspension (CI; Fig. 2). At the genus level, growth stage-dependent dynamics were observed using both CI and CD methods. *Prevotella*, present at 4 (d20), 30 (d61), 25 (d116), and 12% (d174) at the four growth stages, was the most abundant genus based on the CI methods. However, it occupied only a small proportion (<4%) of total cultured microbiota at these stages based on CD methods. Escherichia, Streptococcus, and *Lactobacillus* were the top three genera with 27, 26, and 12% average relative abundance, respectively, as detected by the CD methods (Fig. 2). Other genera such as *Megasphaera*, *Acidaminococcus*, *Bacillus*, *Mitsuokella*, and *Prevotella* were also observed with an average relative abundance of 7, 5, 3.5, 2.5, and 2.2%, respectively (Fig. 2). Some of the abundant genera revealed by the CI method, such as p-2534-18B5_unclassified (lactation stage), *Clostridiaceae_unclassified* (growing and finishing stage), *Ruminococcaceae_unclassified*, and YRC22 (finishing stage), were not recovered using our culturing system (<0.5%; Fig. 2). However, *Bacillus*, barely detectable by CI methods, was observed at growing and finishing stages with a relative abundance of 5 and 7.3%, respectively (Fig. 2). Overall, the genera *Prevotella*, *Megasphaera*, *Lactobacillus*, Streptococcus, *Anaerovibrio*, and *Bacteroides* were well-detected dominant swine gut microbiota by both CD and CI methods.

### (iii) A reference heatmap toward culturing specific bacterial ASVs of interests in pigs.

**FIG 3:**
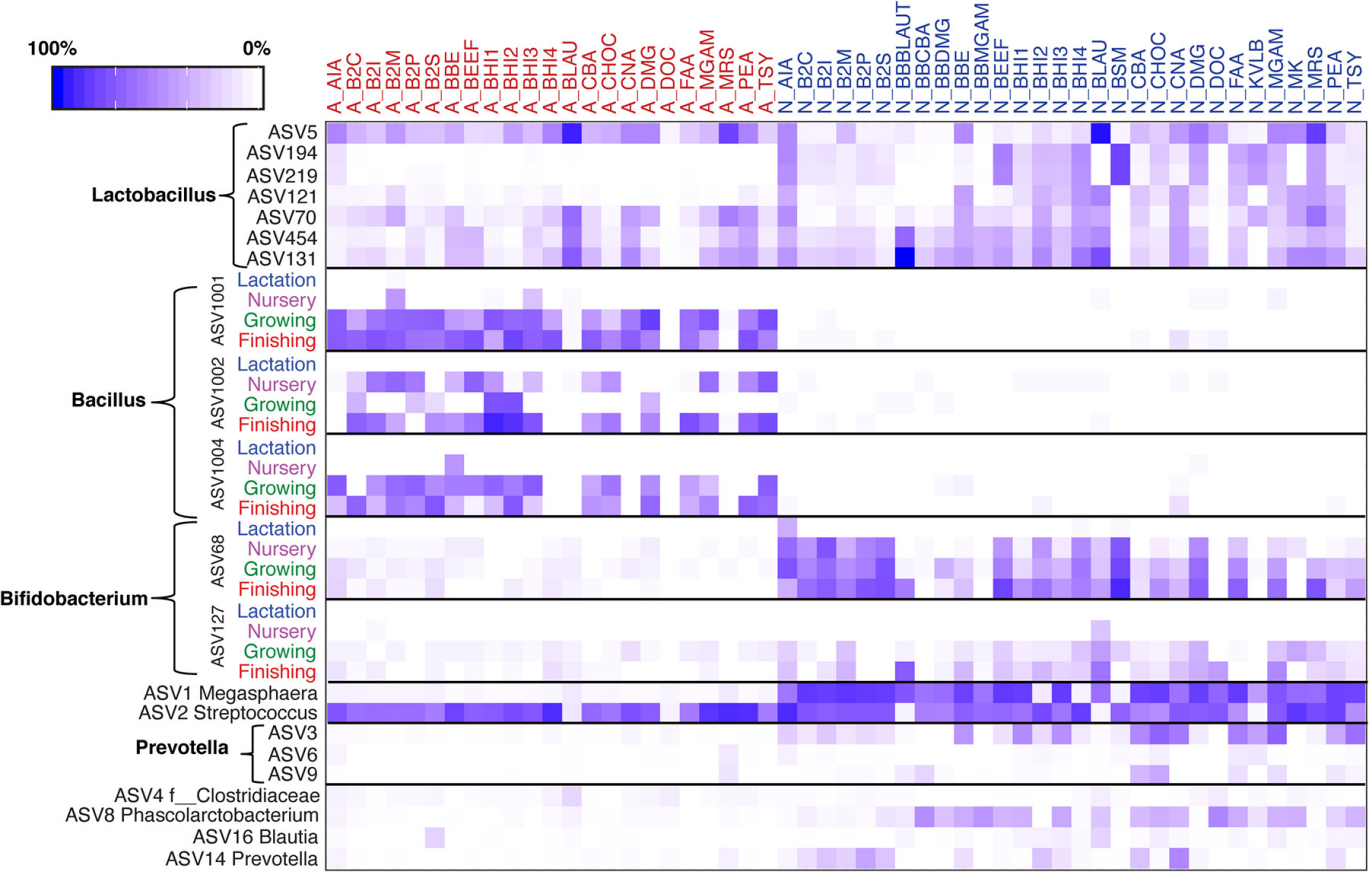
Heatmap of enriched specific bacterial ASVs of interest in pigs under each of the 53 culture methods (A, red, aerobic condition; N, blue, anaerobic condition). The average relative abundances of enriched ASVs associated with *Lactobacillus*, *Prevotella*, and several dominant ASVs (ASV1 *Megasphaera*, ASV2 Streptococcus, ASV4 *Clostridiaceae_unclassified*, ASV8 *Phascolarctobacterium*, ASV16 *Blautia*, and ASV14 *Prevotella*) are shown in the heatmap.

One major goal of this study was to provide a reference method to culture specific bacterial taxa of interest from pigs at different growth stages. To this end, we developed a reference heatmap that includes different bacterial ASVs and their optimal growth conditions (Fig. 3). We defined the culturability of different bacterial ASVs into four categories: easy, moderate, difficult, and yet to be cultured, with a relative abundance of >10, 1 to 10, <1, and 0%, respectively. We describe the following strategies to culture different groups of bacteria from pigs, which may be of interest (e.g., potential probiotics, abundant taxa, core microbiota, and key bacteria) in the following sections.

Potentially beneficial bacteria in pigs include *Lactobacillus*, *Bacillus*, and *Bifidobacterium.* Species of *Lactobacillus* are usually cultured with MRS medium under anaerobic conditions. In our study, MRS agar enriched a large number of *Lactobacillus* bacteria such as ASV5, ASV194, ASV219, ASV121, ASV70, ASV454, and ASV131 (Fig. 3). However, MRS was not the best culture medium for any of them, except for ASV121. For instance, ASV5, ASV70, ASV454, and ASV131 could be enriched by *Blautia* selection medium (BLAU) to 43, 4, 4, and 12%, respectively, regardless of oxygen conditions. Additional blood bottle (BB) preenrichment (BBBLAUT) cultured ASV131 and ASV454 to 65 and 7%, respectively. *Bifidobacterium* selective medium (BSM) under anaerobic conditions was the best option, recovering ASV194 and ASV219 to 18% for both (Fig. 3). Two culture media, BLAU and BSM, were more efficient than MRS in recovering *Lactobacillus* at higher relative abundances.

*Bacillus* ASVs such as ASV1001, ASV1002, and ASV1004 were cultured by a range of BHI-based agars such as BHI1, BHI2, BHI3, B2I, and B2S but only from feces of adult pigs and under aerobic conditions (Fig. 3). Low-abundance *Bifidobacterium* ASV68, an oxygen-sensitive bacterium, was enriched on anaerobic BSM agar up to 11, 17, and 36%, using nursery-, growing-, and finishing-sourced feces, respectively (Fig. 3). Cooked meat agar (BEEF) under anaerobic conditions raised recovery of ASV68 to 18% using finishing-period feces. Additionally, diluted BHI supplemented with substrates such as serum or inulin (B2S and B2I) also enriched ASV68. Another *Bifidobacterium* (ASV127) was favorably enriched on anaerobic agars BLAU, BBBLAUT, deoxycholate agar (DOC), and modified Gifu-anaerobic agar (MGAM) (at 5, 9, 1.5, and 3%, respectively) using finishing pig samples (Fig. 3).

### (iv) Abundant bacterial ASVs in pigs.

The culturability of the top 10 most abundant bacterial ASVs in pigs based on our previous study (5) is shown in Fig. 3. *Megasphaera* (ASV1) is the most abundant bacterium in the swine gut during nursery, growing, and finishing stages and can be enriched in BHI1, B2P (diluted BHI with pectin), BEEF, and diluted MGAM (DMG) (up to 40, 50, 50, and 45%, respectively). The second most abundant bacterial member, ASV2, classified within Streptococcus, can be recovered by a wide range of culture methods under aerobic and anaerobic conditions. *Actinomyces* isolation agar (AIA), BHI4, and BHI2 could exclusively enrich ASV2 to over 70%. Other rich media, MGAM, phenylethyl alcohol agar (PEA), and CNA, were also good options for culturing Streptococcus (Fig. 3). Aside from ASV1 and ASV2, other abundant ASVs belonging to *Prevotella* showed greater variation in culturability. For example, ASV3, although not recovered during lactation, showed moderate culturability after nursery stage under anaerobic conditions, whereas ASV6 and ASV9 were difficult to culture throughout the study period. Other ASVs (ASV4, ASV8, ASV16, and ASV14) in the top 10 list were moderately recovered by variant culturing methods (Fig. 3). Based on our definition of culturability, among the top 100 ASVs detected in CI profiling, 21 were easily culturable, 27 were moderately culturable, 46 were difficult to culture, and 6 were yet to be cultured. The top 100 ASVs detected as having the “best” culturability using CD are illustrated in Table S2.

10.1128/mSystems.00477-21.7 TABLE S2 The best culture conditions and feces origin for the top 100 ASVs (unclassified taxa were removed) in the swine gut microbiome detected by culture-independent methods. Download Table S2, DOCX file, 0.02 MB. Copyright © 2021 Wang et al. 2021 Wang et al. https://creativecommons.org/licenses/by/4.0/ This content is distributed under the terms of the Creative Commons Attribution 4.0 International license.

### (v) Core bacterial ASVs in pigs.

We previously identified 69 core bacterial ASVs (5). Based on our definition, these core members needed to be detected at all 16 time points during the four growth stages. Using our approaches, we were able to culture 63 of the 69 core ASVs. In addition, culture-enriched profiling revealed more bacterial ASVs that might belong to the “missing core microbiota,” i.e., those that were present at specified time points but were missed by the CI methods due to their lower relative abundance and shallow sequencing depth. For example, ASV56, classified within *Bacteroides*, was not included as a core member given that it was not detected at the end of nursery (d61), growing (d116), or finishing stage (d174; Fig. S2b). However, the CD approaches detected this ASV at all these time points (Fig. S2c). A total of 83 such ASVs were detected (Fig. S2d).

### Factors affecting swine gut microbiota cultivation.

We next investigated the effects of different factors such as medium, donor growth stage, and oxygen conditions using permutational multivariate analysis of variance (PERMANOVA; permutations = 999; see Table S3 in the supplemental material). Oxygen ranked as the top driver (F-score_mean_ = 142.1; *R*^2^_mean_ = 0.12; *P* value < 0.001) and greatly shaped the culturable community. The growth stage of donor pigs was the second influencing factor (F-score_mean_ = 35.51; *R*^2^_mean_ = 0.09; *P* value < 0.001) followed by medium type (F-score_mean_ = 10.64; *R*^2^_mean_ = 0.27; *P* value < 0.001).

10.1128/mSystems.00477-21.8 TABLE S3 PERMANOVA of factors such as medium, oxygen, and donor pig age affecting swine culturomics. Download Table S3, DOCX file, 0.02 MB. Copyright © 2021 Wang et al. 2021 Wang et al. https://creativecommons.org/licenses/by/4.0/ This content is distributed under the terms of the Creative Commons Attribution 4.0 International license.

In addition, we performed Random Forest analysis to determine the top 30 oxygen- (Fig. S3a) and donor stage-associated members (Fig. S3b). ASVs such as ASV109 (*Clostridium*), ASV17 (*Collinsella*), ASV1 (*Megasphaera*), ASV121 (*Lactobacillus*), ASV68 (*Bifidobacterium*), and ASV100 (*Acidaminococcus*) were ranked as top ASVs susceptible to oxygen, while ASV7 (Escherichia), ASV1001 (*Bacillus*), ASV1003 (*Bacillaceae*), ASV95 (Streptococcus), and ASV1002 (*Bacillus*) were enriched in the aerobic environment (Fig. S3a).

10.1128/mSystems.00477-21.3 FIG S3 Random Forest and boxplots showing the top 30 bacterial ASVs differentiating aerobic and anaerobic conditions. Red indicates aerobic condition; blue indicates anaerobic condition (a). Categorical Random Forest and boxplots showing the top 30 stage-associated bacterial ASVs. Lactation, nursery, growing, and finishing stages are shown in red, green, blue, and purple, respectively (b). Download FIG S3, PDF file, 1.6 MB. Copyright © 2021 Wang et al. 2021 Wang et al. https://creativecommons.org/licenses/by/4.0/ This content is distributed under the terms of the Creative Commons Attribution 4.0 International license.

**FIG 4:**
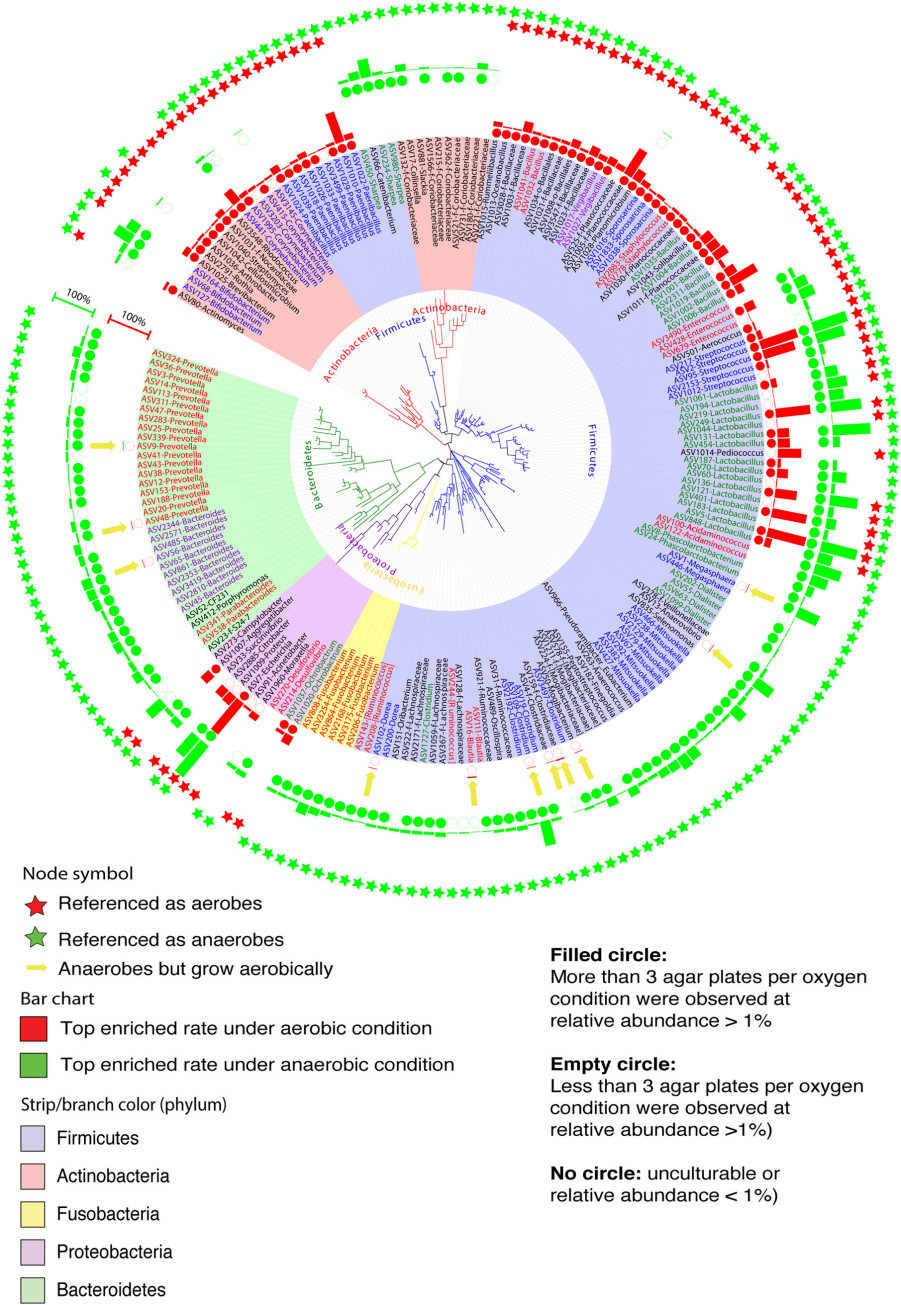
Phylogenetic tree analysis showing the top 200 bacterial ASVs detected on agar plates under both aerobic and anaerobic conditions. The rings of bar charts indicate the relative abundance from the best culture conditions under aerobic (red bar) or anaerobic (green bar) conditions. The rings of circles showing the degree of culturability (filled circles, more than three agar plates per oxygen condition were observed at relative abundance of >1%; empty circles, fewer than three agar plates per oxygen condition were observed at relative abundance of >1%; no circles, yet to be cultured or relative abundance of <1%). Rings of stars showing traditional classification of these ASVs as aerobes (red) or anaerobes (green). The innermost clades and labels were colored by phyla.

Interestingly, we observed some exceptions of anaerobes and aerobes considered by conventional knowledge. Among the 200 most easily cultured ASVs (Fig. 4), 122 were associated with 50 different genera from the *Firmicutes* phylum, known as either strict anaerobes or facultative anaerobes. Consistently, certain genera in this phylum, such as *Clostridium*, *Mitsuokella*, *Dorea*, and *Acidaminococcus*, considered strict anaerobes (28–31), grew only under anaerobic conditions. Members of the Streptococcus and *Lactobacillus* genera, known as facultative anaerobes (32, 33), were enriched to approximately 90% relative abundance regardless of oxygen conditions in our study. Species of genus *Bacillus*, such as B. cereus, B. coagulans, and B. muralis, are generally recognized as aerobes or facultative anaerobes (34). ASVs classified within these species were enriched aerobically. However, exceptions were observed in members associated with *Prevotella* and *Bacteroides* in the *Bacteroidetes* phylum. For example, although bacterial ASVs such as ASV9 (*Prevotella*), ASV48 (*Prevotella*), and ASV56 (*Bacteroides*) are generally considered anaerobes, they can grow aerobically. Other examples are bacterial ASVs associated with *Dorea* (ASV102), *Blautia* (ASV16), and *Clostridiaceae_unclassified* (ASV159), known as anaerobes. These bacteria could still be cultured to a relative abundance of 1 to 3% in the presence of oxygen. Similarly, nine ASVs from the genus *Paenibacillus*, traditionally known as facultative anaerobes, grew only aerobically (Fig. 4). The conditions for maximum growth of these 200 ASVs under aerobic and anaerobic conditions on different media are listed in Fig. S4.

10.1128/mSystems.00477-21.4 FIG S4 Heatmap showing the culturability of the top 200 culturable bacterial ASVs (*y* axis) under 53 culture methods (*x* axis). The culturability of different bacterial ASVs was classified into four categories: easy, moderate, difficult, and yet to be cultured, with a relative abundance of >10, 1 to 10, <1, and 0%, respectively. Hierarchical clustering dendrograms were performed on both the *x* axis and *y* axis. Download FIG S4, PDF file, 0.4 MB. Copyright © 2021 Wang et al. 2021 Wang et al. https://creativecommons.org/licenses/by/4.0/ This content is distributed under the terms of the Creative Commons Attribution 4.0 International license.

Random Forest also disclosed stage-associated bacterial members (Fig. S3b). For instance, ASV7 (Escherichia coli), ASV249 (*Lactobacillus*), and ASV109 (*Clostridium*) noticeably increased at the end of lactation stage. ASV95 (Streptococcus) and ASV848 (*Lactobacillus*) increased during nursery stage. While ASV1003 (*Bacillaceae unclassified*), ASV1004 (*Bacillus*), ASV70 (*Lactobacillus*), ASV428 (*Enterococcus*), and ASV238 (*Mitsuokella*) were associated with growing stage. ASV1002 (*Bacillus*), ASV1008 (*Bacillales*), ASV68 (*Bifidobacterium*), and ASV127 (*Bifidobacterium*) were prominent members from samples collected at the end of finishing stage (Fig. S3b).

We next focused on the basal medium MGAM and its derived cultures (e.g., those with additional blood bottle [BB] preenrichment, dilution, and antibiotic supplementation) to elucidate the effects of other culturing factors. For basal MGAM and derivatives DMG (10× dilution of MGAM) and BLAU (MGAM with sulfamethoxazole and ciprofloxacin), and their anaerobic BB preenrichment versions (BBDMG, BBMGAM, and BBBLAU), antibiotics (analysis of similarity [ANOSIM]: *R* = 0.8, *P* = 0.001), oxygen (*R* = 0.2, *P* = 0.001), and BB preenrichment (*R* = 0.1, *P* = 0.01) were the major drivers of culture profiling (Fig. S5a, b, and c). The dilution factor did not tend to affect the overall cultured microbiota (Fig. S5d). Random Forest analysis showed that antibiotics (sulfamethoxazole and ciprofloxacin) used in BBBLAUT and BLAU enriched members of *Lactobacillus* ASV131 and ASV454, likely by reducing the competitive growth of ASV7 (Escherichia), ASV100 (*Acidaminococcus*), and ASV663 (*Dialister*; Fig. S5e). Additional BB preenrichment without antibiotic supplements enriched ASV224 (*Mogibacteriaceae*), ASV428 (*Enterococcus*), ASV966 (*Eubacterium*), ASV921 (*Ruminococcaceae*), and ASV2129 (*Mitsuokella*) but reduced the growth of ASV2 and ASV382. One of the most abundant ASVs, *Lactobacillus* ASV5, was enriched on BLAU (Fig. S4 and S5e).

10.1128/mSystems.00477-21.5 FIG S5 Bray-Curtis dissimilarity-based principal-coordinate analysis (PCoA) plots show culture-enriched molecular profiling of the swine gut microbiota using MGAM-based media (MGAM, DMG, BLAU, BBDMG, BBMGAM, and BBBLAUT) to study the effects of culturing conditions such as antibiotics (a), blood culture bottle preincubation (b), oxygen condition (c), and medium types (d) on enriched bacteria. Biological replicates were labeled under each point. Biological replicates were labeled under each symbol. Panel e shows relative abundances of the top 30 ASVs associated with different culture factors, as selected by Random Forest under all six MGAM-based conditions. Download FIG S5, PDF file, 1.3 MB. Copyright © 2021 Wang et al. 2021 Wang et al. https://creativecommons.org/licenses/by/4.0/ This content is distributed under the terms of the Creative Commons Attribution 4.0 International license.

### Cooccurrence networks revealed by culture methods.

**FIG 5:**
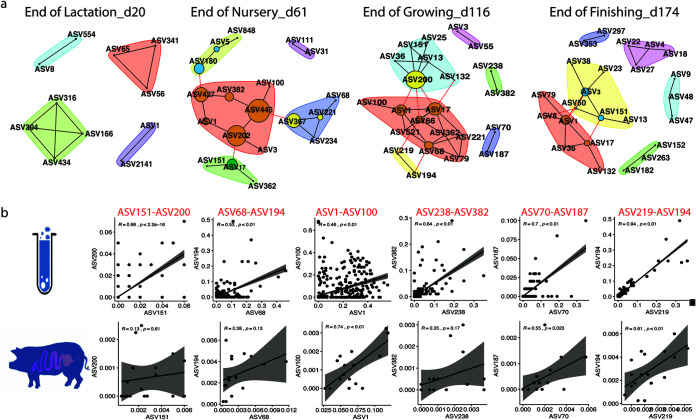
Shared cooccurrence networks disclosed by SparCC (|*R*| > 0.4, *P* < 0.05) in both culture-independent (CI) and -dependent (CD) models at each time point (a). The SparCC algorithm within the Mothur software package was used to construct bacterial pairwise correlations in both the culture-enriched molecular profiling and live pig models. Correlations with coefficiency over 0.4 or less than −0.4 (*P* value less than 0.05) were included for downstream network display. Age-dependent ASVs with cooccurrence that were shared in both culture and live animals were clustered based on the Girvan-Newman algorithm. Sizes of nodes were determined by the betweenness-based centrality. Correlations of six pairs of cooccurring bacterial ASVs on d116 are shown in both CD (upper panel) and CI (lower panel) models (b). The six pairs of ASVs are ASV200 (*Dorea*)-ASV151 (*Oribacterium*), ASV68 (*Bifidobacterium*)-ASV194 (*Lactobacillus*), ASV1 (*Megasphaera*)-ASV100 (*Acidaminococcus*), ASV238 (*Mitsuokella*)-ASV382 (*Mitsuokella*), ASV70 (*Lactobacillus*)-ASV187 (*Lactobacillus*), and ASV219 (*Lactobacillus*)-ASV194 (*Lactobacillus*).

We next asked whether culture enrichment can be used as an *in vitro* model to reveal bacterial cooccurrence based on networks constructed from culture-independent microbiota data collected from the 18 pigs described previously (5). We constructed a cooccurrence network using the culture-enriched molecular profiling data from the three pigs at each of the four time points and compared them to their counterpart networks based on CI methods from the 18 pigs used in our previous study (5). A total of 8,521, 4,676, 6,448, and 6,670 pairs of correlated ASVs were observed from pigs on d20, d61, d116, and d174, respectively (Table 3). Culture-enriched molecular profiling disclosed 125, 217, 284, and 437 pairs of correlated ASVs, respectively, with most of them showing positive correlations. After comparing the CI and CD methods, 11, 19, 33, and 25 pairs of shared cooccurring ASVs were detected in the networks at the four stages (Fig. 5a). Positive correlations of these pairs were observed from both CI- and CD-based networks. Taking the networks on d116 as an example, scatterplots of six pairs of cooccurring ASVs (i.e., ASV151-ASV200, ASV68-ASV194, ASV1-ASV100, ASV238-ASV382, ASV70-ASV187, and ASV219-ASV194) in both culture models and pigs showed consistent positive relationships (Fig. 5b).

**TABLE 3:** Pairwise correlations among bacterial ASVs detected by SparCC (|*R*| > 0.4; *P* < 0.05) in culture-dependent (CD) and -independent (CI)^*a*^ microbiome data at each time point

Correlations, pairs	d20	d61	d116	d174
CI,^*a*^ swine gut (|*R*| > 0.4; *P* < 0.05)	8,521	4,676	6,448	6,670
Positive (*R* > 0.4)	4,658	2,236	3,438	3,510
Negative (*R* < −0.4)	3,863	2,440	3,010	3,160
CD, culturomics (|*R*| > 0.4; *P* < 0.05)	125	217	284	437
Positive (*R* > 0.4)	123	194	270	396
Negative (*R* < −0.4)	2	23	14	41
Shared in CI and CD	11	19	33	25

a The three pigs used for our primary bacterial culturing (CD) are among the 18 pigs whose gut microbiotas have been extensively characterized in a previous study (5). The cooccurrence network was constructed using the gut microbiota data from the 18 pigs.

### Bacterial isolation and identification from pigs.

One major goal of this study was to provide preliminary guidance to isolate certain bacteria of interest from pigs, which will be critical in establishing causal relationships between the gut microbiota and pig health. Such knowledge will be useful for probiotic development and pathogen identification. Also, to determine if the culture enrichment approach (i.e., the bacterial cultivation heatmap) could be used to guide bacterial isolation, we performed a second-round bacterial isolation experiment.

On average, 9, 26, and 18 bacterial ASVs with a relative abundance of over 1% from each agar plate were detected using lactation, growing, and finishing feces, respectively, when all the colonies from these plates were pooled and sequenced (Table 4). Among these ASVs, 6, 12, and 12 were isolated and identified by 16S rRNA gene full-length sequencing, respectively, suggesting that the bacteria enriched by certain cultivation methods having relative abundances greater than 1% are likely isolatable. Moreover, bacteria of some rare ASVs (with relative abundances less than 1%) were also isolated. We then validated the isolated bacteria under specific culture conditions. Consistent with previous findings, a great number of *Lactobacillus* (23% of total colonies), Streptococcus, *Bifidobacterium*, *Corynebacterium*, *Clostridium*, and *Aerococcus* were isolated from MGAM agars under anaerobic conditions. *Prevotella*, *Clostridium*, Proteus, and *Megasphaera* were isolated using anaerobic *Bacteroides* bile esculin agar (BBE).

**TABLE 4:** ASVs in colony mixtures detected by 16S rRNA amplicon sequencing and/or actual ASVs that were isolated^*a*^

Stage and isolation method^*d*^	ASVs^*b*^^,^^*c*^
Lactation feces	
16S	**ASV428, ASV1009, ASV7, ASV2, ASV109, ASV679**, **ASVK3**, **ASVK2**, ASV4050
Isolated	**ASV428, ASV1009, ASV7, ASV2, ASV109, ASV679**, ASV75, ASV70, ASV3569, ASV3200, ASV121, ASV219, ASV136, ASV5, ASV3958

Growing feces	
16S	**ASV1009, ASV127, ASV131, ASV136, ASV187, ASV194, ASV2, ASV219, ASV3686, ASV5, ASV70, ASV95, ASVK1**, ASV1001, ASV1002, ASV1003, ASV1005, ASV1008, ASV1011, ASV1012, ASV1016, ASV1034, ASV164, ASV2070, ASV4066, ASV7
Isolated	**ASV1009, ASV127, ASV131, ASV136, ASV187, ASV194, ASV2, ASV219, ASV3686, ASV5, ASV70, ASV95,** ASV121, ASV1020, ASV109, ASV4050, ASV428, ASV3569, ASV3733, ASV249, ASV1023, ASV679, ASV3200, ASV3030

Finishing feces	
16S	**ASV2, ASV5, ASV68, ASV3686, ASV194, ASV219, ASV70, ASV127, ASV187, ASV131, ASV121, ASV136,** ASV7, ASV164, **ASVK4**, **ASVK5**, ASV1009, ASV95
Isolated	**ASV2, ASV5, ASV68, ASV3686, ASV194, ASV219, ASV70, ASV127, ASV187, ASV131, ASV121, ASV136,** ASV3569

a 16S rRNA amplicon sequencing over 1% from each agar plate.

b Shared ASVs between “16S” and “isolated” are highlighted in bold.

c These ASVs or isolated strains were classified into g_Proteus (ASV1009), f_*Bacillaceae* (ASV1003, ASV1023), f_*Clostridiaceae* (ASV75), f_*Enterobacteriaceae* (ASV3030), f_*Planococcaceae* (ASV1005, ASV1011), g_*Aneurinibacillus* (ASV3569), g_*Bacillus* (ASV1001, ASV1002), g_*Bifidobacterium* (ASV127, ASV164, ASV68), g_*Clostridium* (ASV109, ASV3958), g_*Corynebacterium* (ASV3686), g_*Enterococcus* (ASV428, ASV679), g_Escherichia (ASV7, ASVK4, ASVK5), g_*Lactobacillus* (ASV121, ASV131, ASV136, ASV187, ASV194, ASV219, ASV249, ASV3733, ASV5, ASV70), g_*Morganella* (ASVK2), g_*Ochrobactrum* (ASV1020), g_*Peptostreptococcus* (ASV4050), g_*Providencia* (ASV3200), g_*Sporosarcina* (ASV1016, ASV2027), g_Streptococcus (ASV2, ASV95, ASVK1, ASVK3), and o_*Bacillales* (ASV1008, ASV1034, ASV4066).

d In the second round of bacterial cultivation, fresh feces from lactation, growing, and finishing pigs were used to prepare microbial solutions and to plate on aerobic BHI2 and PEA and anaerobic B2I, BBE, BEEF, BSM, MGAM, and PEA. On each plate, 10 colonies were randomly picked for near-full-length 16S rRNA Sanger sequencing. The rest of the colonies on these plates were pulled and sequenced by 16S rRNA amplicon sequencing.

**FIG 6:**
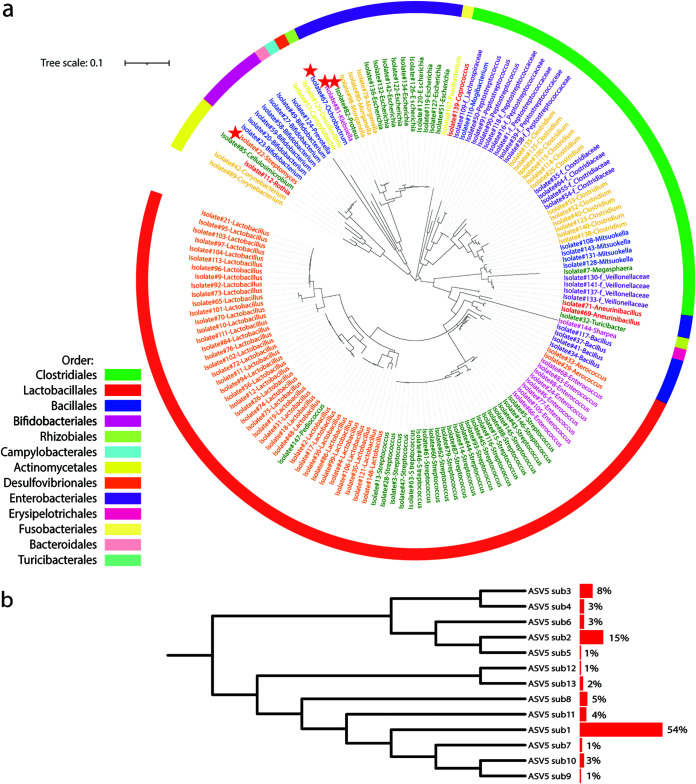
(a) Phylogenetic tree analysis of 148 different bacterial taxa based on 16S rRNA gene V3-V7 hypervariable regions. Colonies were picked from eight agars (aerobic BHI2 and PEA and anaerobic B2I, BBE, BEEF, BSM, MGAM, and PEA). Isolates were identified by colony-PCR using a 16S rRNA gene primer set, 27f and 1492r. PCR amplicons were validated by gel electrophoresis and cleaned up for Sanger sequencing. Forward and reverse sequences were aligned to obtain near-full-length 16S rRNA gene contigs. All contigs were truncated to 16S rRNA gene V3-V7 (F, 5′-TACGGRAGGCAGCAG-3′, and R, 5′-GTAGCRCGTGTGTMGCCC-3′) before phylogenetic tree analysis using the Qiime2 program. The outermost ring was colored by orders. The clades of the tree were colored by genus. Node with a red star possesses a less than 95% similarity to the NCBI BLAST online database, indicating a potentially novel strain in the swine gut microbiome. (b) Phylogenetic tree of 13 sub-ASVs classified with ASV5 *Lactobacillus*. Side bar charts showing the contribution of each sub-ASV to the total ASV5 colony numbers.

Collectively, we Sanger sequenced a total of 1,299 colonies, which were classified into 148 different bacterial ASVs belonging to 28 genera and six phyla based on 16S rRNA gene (V3-V7 region) sequences as shown in the phylogenetic tree (Fig. 6a). These ASVs include isolates affiliated with potentially beneficial taxa (e.g., *Lactobacillus*, Streptococcus, *Bifidobacterium*, *Pediococcus*, and *Bacillus*), opportunistic pathogens (e.g., Escherichia, Campylobacter, and *Clostridium*), and other members such as *Megasphaera*, *Turicibacter*, *Mitsuokella*, *Lachnospiraceae*, f_*Fusobacterium*, f_*Veillonellaceae*, *Rothia*, Proteus, Klebsiella, and *Sharpea.* In addition, four bacterial ASVs, having less than 95% similarity in their full 16S rRNA gene sequences compared to other bacteria in the NCBI BLAST online database (2.10.1+), are potentially novel bacterial taxa in the swine gut (12, 35, 36).

Of note, Sanger sequencing of the near-full-length 16S rRNA gene in bacterial isolates disclosed inadequate resolutions by using the MiSeq platform targeting only the V4 region. For example, we intensively cultured one of the most abundant bacterial ASVs (ASV5) in pigs affiliated with *Lactobacillus* using BLAU and MGAM media under anaerobic conditions according to the heatmaps described above. We cultured 100 isolates with 100% similarity in the 16S rRNA gene V4 region compared to bacterial ASV5 using a culture-independent method. However, the near-full-length sequence of the 16S rRNA gene showed 13 sub-ASVs from these isolates (Fig. 6b). Among these sub-ASVs, isolates with ASV5-sub1 accounted for over half of the total ASV5 isolates.

## DISCUSSION

Remarkable achievements in the field of human culturomics have vastly enhanced our understanding of human gut microbiota. However, little effort has been made regarding the cultivation of the swine gut microbiota despite recent progress in swine gut microbiome research using CI approaches. In this study, we conducted large-scale culturing of swine gut microbiota using 53 methods. This investigation expanded our knowledge of swine gut microbiota diversity and provided guidance for future research targeting specific bacteria of interest in pigs.

At each of the four growth stages, approximately half of the bacterial ASVs were recovered using our culture systems. Not surprisingly, the percentage of culturable bacteria from pigs increased at a higher classification level (73 to 81% at genus levels or 65 to 83% at family levels). Our data agreed with human culturomic studies that reached similar recovery rates. Using 10 different anaerobic culturing methods (e.g., M9, GMM, and MAGM), Rettedal et al. recovered 50% of ASVs found in culture-independent profiles (17). Of note, most of the nonculturable ASVs were less than 2%. In a recent study, Ito and colleagues recovered 61% of the total ASVs in fecal samples using a total of 26 culturing media (19). Lau and colleagues (16) cultured an average of 76% of bacterial ASVs by using 66 culturing conditions. Interestingly, when low-abundant ASVs (<0.1%) were removed, the recovery rate went up to 95%. Consistently, in our project, the recovery rates went up to 87 and 99% on average when removing low-abundant ASVs lower than 0.1 and 1%, respectively (see Table S4a in the supplemental material), suggesting that these rare bacteria either were inactive (e.g., dormant), were not competitive on culture medium agar plates, or required otherwise special culture conditions.

10.1128/mSystems.00477-21.9 TABLE S4 Recovery rates, i.e., proportions of cultivable ASVs in the culture-independent group, are shown at each time point, with two thresholds set at 0.001 and 0.01 for low-abundant microbes (a). Percentages of bacterial families under anaerobic and aerobic conditions at four time points (b). Download Table S4, DOCX file, 0.01 MB. Copyright © 2021 Wang et al. 2021 Wang et al. https://creativecommons.org/licenses/by/4.0/ This content is distributed under the terms of the Creative Commons Attribution 4.0 International license.

Our culture-independent next-generation sequencing-based approach showed that pigs harbor a large number of different bacterial taxa. Crespo-Piazuelo and colleagues revealed 1,261 operational taxonomic units (OTUs) from 18 phyla and 101 genera in the swine gut (37). In another study, Holman and Chenier detected 1,281 different OTUs from 15 different bacterial phyla and 65 genera (38). In our previous study, we demonstrated an average of 1,178 bacterial ASVs ranging from 809 to 1,156 at 16 time points across the four growth stages in 18 pigs (5). In this study, CI methods detected 378, 482, 565, and 555 ASVs at each stage, respectively. This work significantly expanded our view of the microbial richness in pigs. A total of 244, 411, 507, and 510 new bacterial ASVs undetected when using the CI methods were observed at the four time points by using CD methods. Adding CD and CI yielded 622, 893, 1,072, and 1,065 bacterial ASVs. The reasons why CI methods failed to detect these new ASVs likely include their relatively low abundance below the detection limits of sequencing depth, incomplete lysis of bacterial cells during DNA extraction, or unfavorable amplification during PCR.

In addition, 16S rRNA gene sequences of the 100 bacterial isolates affiliated with ASV5 *Lactobacillus*, from different growth conditions, showed that although these isolates were identical based on the V4 region, they are classified into 13 sub-ASVs according to their V3-V7 hypervariable regions. This suggested that the swine gut microbiota might be much more diverse than what we anticipated based on conventional microbiota sequencing targeting only the V4 region of the 16S rRNA gene (39, 40).

Some of the bacterial ASVs missed by CI methods are possibly core members. One example is ASV56, which was classified within the genus *Bacteroides*, which are Gram-negative, rod-shaped obligate anaerobes and a known dominant genus in humans. In pigs, the genus *Prevotella* is more dominant than *Bacteroides*, especially after weaning. Members of *Bacteroides* never became dominant taxa at any of the growth stages of pigs, although they were among the more abundant genera in nursing piglets, suggesting that they might be involved in the digestion of milk-derived proteins, lactose and galactose (41, 42). This was in agreement with human studies where *Bacteroides* (or *Bacteroidetes*) was more abundant in people consuming a westernized diet containing high sugar, protein, and animal-derived products (43, 44), which might explain the low abundance of *Bacteroides* in pigs due to their plant-based diets. In our previous study, *Bacteroides*-associated ASV56 was detected during lactation but disappeared at subsequent stages. Consistently, ASV56 was detected at the end of lactation with a low abundance (0.85%) in this study, but not at any other stage, by the CI methods. Interestingly, we were able to capture this bacterial ASV through the use of BBMGAM culture-enriched molecular profiling method at all four stages, showing a relative abundance up to 24% at the finishing stage. These data suggested that *Bacteroides* may be a core member of the swine gut microbiota; however, its relative abundance decreased to a level that was not detectable by the CI methods at the other stages. Of note, the abundance detected by culture in other stages was comparable to that of the lactation stage, suggesting that *Bacteroides* absolute abundance might not decrease in those stages. Other bacterial taxa may have proliferated more than this genus and outcompeted it to make *Bacteroides* a relatively low-abundant bacterial ASV.

One of the major goals of this study was to provide a reference heatmap to culture certain bacterial taxa of interest from pigs. Although multi-omics studies provide important insights into bacterial functions concerning animal health and disease (45, 46), isolation of bacterial strains and subsequent validation using animal models are the most convincing approach to prove these functions.

Probiotics are of particular interest to scientists and producers as an alternative to replace the use of antibiotics as growth promoters in livestock, a known pathway to produce troublesome antibiotic-resistant microbes that can have deleterious effects upon public health. Common probiotics in the swine industry include *Lactobacillus* and *Bacillus*. *Lactobacillus* plays beneficial roles in gut health, such as regulation of intestinal mucosal immunity, maintenance of gut barrier function, and competition with pathogens for binding sites (47, 48). It has been identified by CI methods as one of the most abundant genera in pigs, even at different niches of the gastrointestinal (GI) tract (26). Members of this genus are relatively easy to culture. Of note, most of the *Lactobacillus* ASVs can grow on the MRS medium, but certain ASVs such as ASV5, ASV194, and ASV219 grow better on other media, suggesting differences in physiological and biochemical properties at the species level. Using MGAM under both anaerobic and aerobic conditions, we successfully recovered hundreds of isolates that classified into 39 different *Lactobacillus* strains based on the 16S rRNA gene sequences (V3-V7 region), which greatly extended the list of probiotic candidates.

The genus *Bacillus* contains certain species that are used as probiotics in pigs due to their antimicrobial activity and ability to form spores, reduce the occurrence and severity of diarrhea in piglets, and modulate the immune system (49). However, such beneficial probiotics have been overlooked in culture-independent approaches, most likely because they form spores, a characteristic that makes DNA extraction more difficult. For example, the *Bacillus* genus was not detected in pigs by culture-independent methods, even in groups supplemented with high doses of *Bacillus* probiotics (50). In our previous characterization of the longitudinal swine gut microbiota in 18 pigs across 16 time points, only one *Bacillus*-related ASV, ranking 2,047 out of a total of 3,254 ASVs, was observed on d90 (0.14% from two pigs) and d116 (0.025% from one pig). Consistently, only two ASVs, ASV2311 (0.012%, lactation) and ASV1001 (0.01%, finishing), were detected by CI methods in this study. However, many more ASVs (4, 18, 18, and 27 at lactation, nursery, growing, and finishing stages, respectively) of *Bacillus* were cultured to a high relative abundance at different stages by the CD approach, suggesting that these members are likely residents of the swine GI tract. As determined by our CD method in Fig. 3, we have successfully isolated four different *Bacillus* strains, including ASV1004 and ASV1002, using aerobic diluted MGAM agar from fecal samples of adult pigs, suggesting that these ASVs are gut residents that were not detected by the CI methods.

While rich media such as tryptic soy agar (TSY), MGAM, Columbia blood agar (CBA), BHI1, chocolate agar (CHOC), and BEEF are used to culture a wider range of bacteria, selective media are designed to select for specific bacteria while hindering the growth of others. Some examples are AIA (for *Actinomycetes*), BBE (for *Bacteroides*), BLAU (potentially for *Blautia*) (17), MRS (for *Lactobacillus*), and BSM (for *Bifidobacterium*). However, some of these media were not efficient in enriching their target bacteria. AIA failed to enrich *Actinomycetes* (<0.05%). Instead, it raised the relative abundance of *Mitsuokella* (belonging to the *Veillonellaceae* family) from initially 1% in feces, as detected by CI (nursery stage), to 45% in total cultured bacterial cells under anaerobic conditions (or from 2 [CI] to 40% [CD] in growing stage feces), and *Bacillus* from 0.02% (CI) to 13% (CD) using finishing stage feces aerobically. A human culturomics project also observed over 10% relative abundance of *Veillonellaceae* by using AIA in an anaerobic environment (16). Notably, CNA was the best culture medium to enrich *Actinomycetes* to 2% using lactation feces under both anaerobic and aerobic conditions in our study. BBE was not able to enrich *Bacteroides* from pigs as it did with humans, where multiple OTUs belonging to *Bacteroides* grew on BBE with high abundance (e.g., *Bacteroides* at 85%) (19), while *Bacteroides* from pigs can potentially grow on BBMGAM and BBCBA under anaerobic condition. This suggests a variation in *Bacteroides* populations between pigs and humans. Instead, BBE greatly improved the relative abundance of *Clostridium* at lactation from 0.45% to 53% aerobically.

Another culture medium that proved to be efficient in growing bacterial species from both pigs and humans is BSM. It enriched *Bifidobacterium* anaerobically from 0.3 (CI) to 18% (CD) on average from pigs, consistent with human culturomics where *Bifidobacteriaceae* were present in over 10% of all cultured organisms (16). BSM under anaerobic conditions proved to be a good culture medium for *Sharpea*, given the 65-fold enrichment from 0.23% to a final proportion of 15%. BLAU agar, a modified MGAM agar supplemented with sulfamethoxazole and ciprofloxacin, greatly enriched *Blautia* (17) as expected. Interestingly, it also recovered several ASVs associated with *Lactobacillus* (90%), such as ASV5.

Despite the remarkably high bacterial diversity and enrichment of certain bacterial taxa resulting from our extensive bacterial cultivation, selection of bacterial culture media and data interpretation need careful consideration. For example, although MRS agar has been widely used to culture *Lactobacillus*, only 55% of bacterial communities grown on this medium were classified as *Lactobacillus*, with Streptococcus and Escherichia contributing to 31% of the total. Therefore, our data suggested that culture-dependent studies aiming to culture *Lactobacillus* need to be validated by sequencing approaches to rule out the non-*Lactobacillus* bacteria. In addition, the relative abundance of the enriched taxa is affected by other factors such as colony size and 16S rRNA gene copy numbers and should be interpreted with caution.

The GI tract is an anaerobic environment enabling the habitation of oxygen-sensitive bacteria. Thus, it was not surprising that a greater diversity and population of ASVs were recovered under oxygen-free conditions at all four time points (51). However, after reassessing the top 200 cultured ASVs, we found several exceptions to conventionally classified anaerobes. For example, *Prevotella* (ASV9) and *Blautia* (ASV16), recognized as strict anaerobes, were observed growing aerobically on MRS and B2S (diluted BHI with starch) agar, respectively. This is consistent with human culturomics reports where members of *Prevotella* and *Blautia* were able to grow aerobically on glutathione-, uric acid-, and ascorbic acid-supplemented Schaedler (GAS) agar (52, 53). ASV159 and ASV56, classified within *Clostridiaceae* and *Bacteroides* from the strict anaerobe list, were observed to grow on CNA and MRS agar (to 3 and 1%, respectively) under aerobic conditions. These data have modified our rigid view of conventional anaerobes and highlighted variations in physiological properties of bacterial ASVs within the same species.

Studies have shown that the gut microbiota of swine is more like that of humans than mice (54). Members of several families such as *Corynebacteriaceae*, *Bacteroidaceae*, *Streptococcaceae*, *Lachnospiraceae*, *Enterobacteriaceae*, and *Erysipelotrichaceae* have been the dominantly enriched taxa in human culturomics studies (16). Consistently, our swine culture-enriched molecular profiling data showed similar culturable profiles. Human *Enterobacteriaceae* and *Streptococcaceae* can grow aerobically on a wide range of media such as TSY, BHI1, and CNA. These members showed similar growth patterns in our investigations of pig fecal samples. These members of *Enterobacteriaceae* and *Streptococcaceae* were able to grow aerobically with an average abundance of 47 and 19%, respectively (Table S4b). In addition, members of *Bacillaceae* from both human and swine feces were remarkably enriched under aerobic conditions (BHI1, MGAM, and DMG in our study; Table S4b). Members of other families, including *Veillonellaceae*, *Coriobacteriaceae*, *Erysipelotrichaceae*, *Bifidobacteriaceae*, and *Bacteroidaceae* belonged to culturable abundant taxa, under anaerobic conditions, in both human (16) and swine studies, particularly this study. Of note, it seems anaerobic BEEF is an exceptional culture medium for both human and swine gut microbiota. It enriched similar culturable bacterial profiles with members from many different families such as *Bifidobacteriaceae*, *Coriobacteriaceae*, *Streptococcaceae*, *Clostridiaceae*, *Veillonellaceae*, *Erysipelotrichaceae*, and *Enterobacteriaceae* (Table S5). Similar culturomic profiles between pigs and humans support the notion that pigs serve as a good model for human biomedical studies.

10.1128/mSystems.00477-21.10 TABLE S5 Anaerobic medium BEEF recovered similar culturable bacterial profiles using both humans’ (16) and pigs’ samples. Download Table S5, DOCX file, 0.01 MB. Copyright © 2021 Wang et al. 2021 Wang et al. https://creativecommons.org/licenses/by/4.0/ This content is distributed under the terms of the Creative Commons Attribution 4.0 International license.

It is worth noting that we did not choose all the colonies from each plate. Due to the labor concerns of our study, we decided to pick 10 clearly isolated colonies per plate (*n* = 10/plate) for processing. Therefore, it is possible that certain bacterial ASVs, which appeared on our plates, may not have been chosen. However, our validation trial, based on the cultivation heatmap, showed that most of the bacterial ASVs with a relative abundance of over 1% on the plates could be cultured by randomly picking such colonies. This suggests that although moderate labor is needed for isolation and identification, our cultivation heatmap does provide guidelines for the selection of certain medium and gas combinations needed to isolate specific bacteria of interest.

The hundreds of bacterial ASVs in pigs do not exist in isolation but interact with one another, with the diet, and with the host in different patterns. Although it is extremely challenging to dissect the interactions between these bacteria due to their complexity and interanimal variation, different models have been constructed to infer the bacterial-bacterial interactions via network analysis (55, 56). A cooccurrence network demonstrates positive or mutualistic interactions in a time- or space-based ecological system (55, 57). These interactions could also be a result of environmental changes or third-party intervention (58). Studying microbial cooccurrence patterns with similar responses to environmental changes could potentially imply the nonrandom community shifts (57).

In this study, we constructed networks at four time points using both CI- and CD-based microbial profiling and discovered shared pairs of bacterial interactions between these two methods. Cooccurrence between ASV1-*Megasphaera* and ASV17-*Collinsella* was detected in both live pigs and culture-enriched molecular profiling at three different stages, indicating either positive or mutualistic relationships in pigs. In addition, ASV1 and ASV17 are dominant swine gut microbes with a high degree of centrality (Fig. 5a), which suggests that these bacterial ASVs might be the keystone taxa that play important roles in the gut and can influence whole-gut ecology shifts (59). Members of *Megasphaera* and *Collinsella* are short-chain fatty acid producers, feeding on animal- and plant-sourced carbohydrates such as lactose, fructose, and starch, which are major components of the swine diet (60–62). *Megasphaera* members are amino acid-metabolizing bacteria of the swine small intestine (63). Genome analysis of *Megasphaera* members indicated that they are potentially diverse amino acid-producing microbes (59, 64). Besides, low dietary fiber intake enhances the growth of *Collinsella* by enabling the utilization of “nonfiber” energy sources such as host glycoprotein (62, 65, 66). This highly flexible metabolic cross-feeding between microbes could explain the potential positive intercommunity relationships that benefit host health (67).

### Conclusions.

Human gut culturomics have detected many new bacterial species, led to the culturing of several most-wanted species, and made it possible to test the functions of certain bacteria of interest. Despite recent advances in swine gut microbiome research, little is known about the culturability of the swine gut microbiota. In this study, we applied 53 culture methods on 12 fecal samples collected from three pigs from four different growth stages to study the culturability of swine gut microbiota and provide guidance for such investigations.

Our data show that approximately 50% of total ASVs and 75% of genera and families were culturable. Together with a culture-independent sequencing-based approach, comprehensive cultivation remarkably expanded our knowledge of swine gut microbiota richness. We also isolated many important bacterial ASVs, including several novel species and important core ASVs, which were overlooked by culture-independent approaches.

Furthermore, we generated a reference heatmap to guide the culturing of specific bacterial groups of interest, such as certain probiotics and dominant bacterial taxa in pigs. We also examined the effects of different factors such as oxygen, growth medium, donor age, antibiotics, and blood bottle culture preenrichment on swine gut microbiota cultivation. By comparing our results to human culturomics, similar patterns were observed which reinforced the idea of similarity between swine and human gut communities.

Finally, network analysis showed shared cooccurrence patterns between the culture-independent microbiota, validating the discoveries made from ecological models developed with *in vivo* data.

## MATERIALS AND METHODS

### Study design and animals.

Three piglets (two females and one male; PIC29*380) were randomly selected from three sows at the University of Arkansas-Division of Agriculture Swine Research Unit. These pigs were identified based on their ear notches at weaning, housed in different pens, and then followed longitudinally for fecal sample collection at different growth stages (from the same three pigs). All piglets were managed as described previously (5). Briefly, the piglets were sow fed until weaning at day (d) 20 and then transferred to a nursery facility, where they were housed with two pigs per pen. On d61, they were transferred to a growing and finishing facility. All pigs were fed with antibiotic-free standard corn soybean meal-based diets that met the National Research Council (NRC, 2012) nutrient requirements (see Table S1 in the supplemental material). Diets were formulated based on growth status, including three nursery, two growing, and two finishing phases. The animal trial was approved by the Institutional Animal Care and Use Committee (IACUC) under protocol 19017. Pigs were managed according to the same approved protocol.

10.1128/mSystems.00477-21.6 TABLE S1 Nutritional composition was formulated based on NRC (2012) nutrient requirements. Diets were formulated based on growth status, including three nursery phases (Np1, Np2, and Np3), two growing phases (Gp1 and Gp2), and two finishing phases (Fp1 and Fp2). Download Table S1, DOCX file, 0.02 MB. Copyright © 2021 Wang et al. 2021 Wang et al. https://creativecommons.org/licenses/by/4.0/ This content is distributed under the terms of the Creative Commons Attribution 4.0 International license.

### Sample collection and culture.

On d20 (end of lactation), d61 (end of nursery), d116 (end of growing), and d174 (end of finishing), fresh fecal samples were collected from these three pigs directly into 50-ml sterile conical tubes by rectal massage. Feces from each sample (0.2 g) were immediately resuspended in 20 ml of sterile physiological saline (0.85 g/liter NaCl supplemented with 1 g/liter thioglycolate) in a sterile WhirlPak filter bag with 0.33-mm pore size and subsequently homogenized in a stomacher (Seward, Cridersville, OH) for 3 min at medium speed (68). Aliquots (50 μl) of the cell suspension were then transferred to a Coy anaerobic chamber (Coy Laboratory Products, Grass Lake, MI, USA; atmosphere of 5% hydrogen, 10% carbon dioxide, and 85% nitrogen) and aerobic biosafety cabinet and incubator for culturing. For anaerobic culturing, agar plates were prepared and maintained anaerobically overnight in the Coy chamber to remove oxygen before plating. A total of 53 culture methods were applied in the first culturing project, and eight culture methods were repeated on samples from different pigs to confirm the efficacy of these isolation methods (Table 1). All agar plates were prepared following the manufacturer’s instructions or as described previously (14, 16, 17). Briefly, aliquots of 50 μl cell suspension were spread plated onto each agar plate and incubated at 37°C for 3 days under either anaerobic or aerobic conditions. With certain methods, blood culture bottles were used for sample preenrichment. From each sample suspension, a 50-μl aliquot was inoculated into blood culture bottles (Bactec Standard Anaerobic/F culture vials [BD, Sparks, MD, USA] supplemented with 4 ml of sheep blood) and incubated at 37°C for 4 days. After incubation, 50 μl from each bottle was spread on a corresponding agar plate and incubated anaerobically for 3 days at 37°C. After incubation, colonies were scraped off from each agar plate by adding 1 ml of 20% glycerol thioglycolate transport medium and were stored under at −70°C before DNA extraction. Colonies from a total of 630 plates were collected including 3 pigs × 4 time points × 52 culture conditions (excluding the culture method of *Blautia* selective medium with a blood bottle preenrichment step [BBBLAUT], *n* = 624) and 2 time points (growing and finishing) × 3 pigs (*n* = 6) with BBBLAUT.

### DNA extraction and MiSeq sequencing.

One aliquot (50 μl) from the WhirlPak filter bag suspensions (12 samples collected from three pigs at four different growth stages; culture-independent technique [CI]) and collected microbial colony mixture from each plate (*n* = 630; culture-dependent technique [CD]) were used for 16S rRNA gene amplicon sequencing as described previously (5). Briefly, DNA was extracted with the bead-based PowerLyzer PowerSoil DNA isolation kit (Qiagen, Germantown, MD, USA) according to the manufacturer’s protocol. DNA quality and quantity were measured on a NanoDrop One^C^ (Thermo Fisher Scientific, Wilmington, DE, USA), and libraries were constructed following the published protocol (12). The bacterial 16S rRNA gene V4 region was amplified for 35 amplification cycles using primers (F, 5′-GTGCCAGCMGCCGCGGTAA-3′, and R, 5′-GGACTACHVGGGTWTCTAAT-3′) with attached barcoded index and Illumina adapters. PCR amplicons were verified by agarose gel electrophoresis for quality and then normalized and purified with the SequalPrep normalization plate kit (Invitrogen, Carlsbad, CA, USA). Normalized amplicons were quantified by a Qubit fluorometer (Thermo Fisher Scientific, Waltham, MA) and then pooled in equal volume. The quality and quantity of the pooled amplicons were measured with an Agilent Bioanalyzer 2100 (Agilent, Santa Clara, CA, USA) and by quantitative RT-PCR, respectively. Illumina MiSeq 2 × 250-bp paired-end sequencing (MiSeq reagent kit v2, 500 cycles, 20% PhiX) was used to sequence pooled amplicons. A mock community was included in each run by combining a defined mixture of microbial genomic DNAs with known proportions (ZymoBIOMICS Microbial Community Standard; Zymo, Irvine, CA, USA). As a quality control, the mock community was processed and analyzed along with the CD and CI samples using the same protocols to control for environmental contamination, sequencing errors, and run-to-run variations.

### Microbiome data analysis.

Raw reads were analyzed using the Quantitative Insights Into Microbial Ecology2 (QIIME2) platform (version 2.4) and Deblur program (13) following the tutorial (https://docs.qiime2.org/2020.11/tutorials/). Briefly, Illumina MiSeq raw forward and reverse reads were imported to the QIIME2 platform and were assembled by “qiime vsearch join-pairs.” Then, “qiime quality-filter q-score-joined” was used to remove low-quality reads (–p-min-quality 30). The retained reads were denoised by Deblur (command: qiime deblur denoise-16S) and trimmed to 240 bases from the 5′ primer end. Deblur-denoised sequences were assigned to amplicon sequence variants (ASVs) at single-nucleotide resolution. For each sample, ASVs less than 10 reads were removed by default (16). All samples were rarefied to the lowest reads, i.e., 4,500 reads, to minimize the effects of sequencing depth on alpha and beta diversity measures using “qiime feature-table rarefy” in Qiime2. Alpha and beta diversity analyses were performed using “qiime diversity alpha” and “qiime diversity beta” commands, respectively.

Bacterial ASVs were classified against the Greengenes reference database (13-8 version) with 99% similarity (69). This sequencing and data analysis pipeline yielded high-quality reads, as suggested by consistent relative abundances of each bacterial ASV from mock communities between different MiSeq runs.

Analysis of similarity (ANOSIM) was performed in QIIME2 by command “qiime diversity beta-group-significance.” Classification-based Random Forest models in R (randomForest packages) were developed to identify bacterial ASVs associated with different culturing factors (70). The Sparse Correlations for Compositional data (SparCC) algorithm (71) within the Mothur software package (72), developed to deal with compositional data, was used to construct age-dependent bacterial pairwise correlations in both the culture-based project (data from the 53 culture conditions) and culture-independent data from live pigs (5). Due to the small sample size (*n* = 3) in the CI group, we applied the microbiota data from all 18 pigs in a previous study (5) for network construction, which included the three pigs used in the culture project. These networks were built based on the SparCC algorithm using the top 300 ASVs from each time point. Correlations with coefficiency over 0.4 or less than −0.4 (*P* value less than 0.05) were included for network display by the igraph package in RStudio software (version 1.2.5019). Age-dependent ASVs with cooccurrence that were shared in culture-enriched molecular profiling and live animals were clustered based on the Girvan-Newman algorithm (73). Sizes of nodes were determined by the betweenness-based centrality (74). Permutational multivariate analysis of variance (PERMANOVA), a distribution-free algorithm working with unbalanced data sets, was performed to disclose factors (e.g., oxygen, age, antibiotics, and blood culture preenrichment) shaping the culturable swine gut microbiota (75). PERMANOVA was performed with the adonis function in the vegan package of R with default settings.

### Bacterial isolation, identification, and phylogenetic tree construction.

A second-round of culturing was conducted using eight selected conditions based on the first-round data (aerobic BHI2 and PEA and anaerobic B2I, BBE, BEEF, BSM, MGAM, and PEA) to isolate a wide range of commensal and beneficial bacteria such as *Lactobacillus*, *Bacillus*, and *Bifidobacterium* and to validate the culture methods developed above. Fresh feces were randomly collected from pigs at lactation, growing, and finishing stages at the same swine farm. These pigs were raised under the same conditions as the three pigs used in the first round of cultivation. We plated these samples onto the eight selected media and randomly picked 10 colonies per plate for full-length 16S rRNA gene Sanger sequencing. The remaining colonies on the plates were pooled and subjected to 16S rRNA gene amplicon sequencing as described above. Colony-PCR was performed using a 16S rRNA gene primer set: 27f forward primer (5′-GAGTTTGATCCTGGCTCAG-3′) and the 1492r reverse primer (5′-TACCTTGTTACGACTT). PCR amplicons were validated by gel electrophoresis and cleaned up using PCR and the Sequencing Reaction Clean-Up 96-well kit (Magnetic Bead System; Norgen Biotek, Thorold, ON, Canada) for Sanger sequencing. Forward and reverse sequences were aligned using the Mothur program to obtain near-full-length 16S rRNA gene contigs. All contigs were truncated to 16S rRNA gene V3-V7 (F, 5′-TACGGRAGGCAGCAG-3′, and R, 5′-GTAGCRCGTGTGTMGCCC-3′) followed by phylogenetic tree analysis in Qiime2 (76). Briefly, representative sequences were aligned using Multiple Alignment using Fast Fourier Transform (MAFFT) in Qiime2 (command: qiime alignment mafft). Then, unconserved sequences and highly gapped columns were removed by command qiime alignment mask. Aligned sequences were further constructed into a phylogenetic unrooted tree using maximum-likelihood nearest-neighbor interchanges with the “qiime phylogeny fasttree” command. Finally, a rooted phylogenetic tree was generated by midpoint rooting and visualized through online software Interactive Tree Of Life (iTOL; version 5.5).

### Availability of data and material.

The data sets generated during and/or analyzed during the current study were deposited in the Sequence Read Archive (SRA) repository under the accession number PRJNA627060.
