# Supplementary figures and images for: Comprehensive Cultivation of the Swine Gut Microbiome Reveals High Bacterial Diversity and Guides Bacterial Isolation in Pigs
Source: mSystems. 2021 Jul 20;6(4):e00477-21. doi: 10.1128/mSystems.00477-21 (PMC8407297; doi:10.1128/mSystems.00477-21)

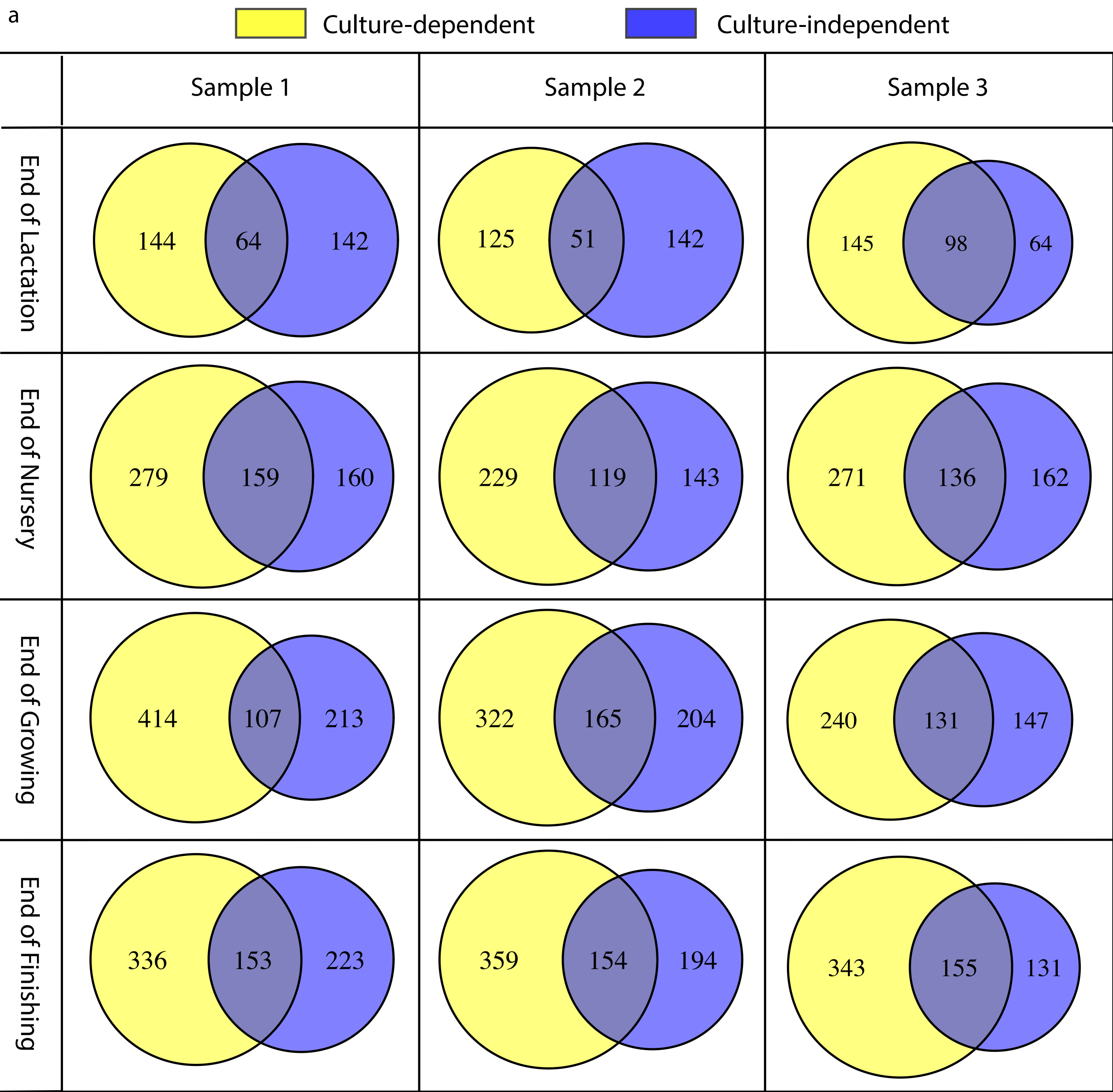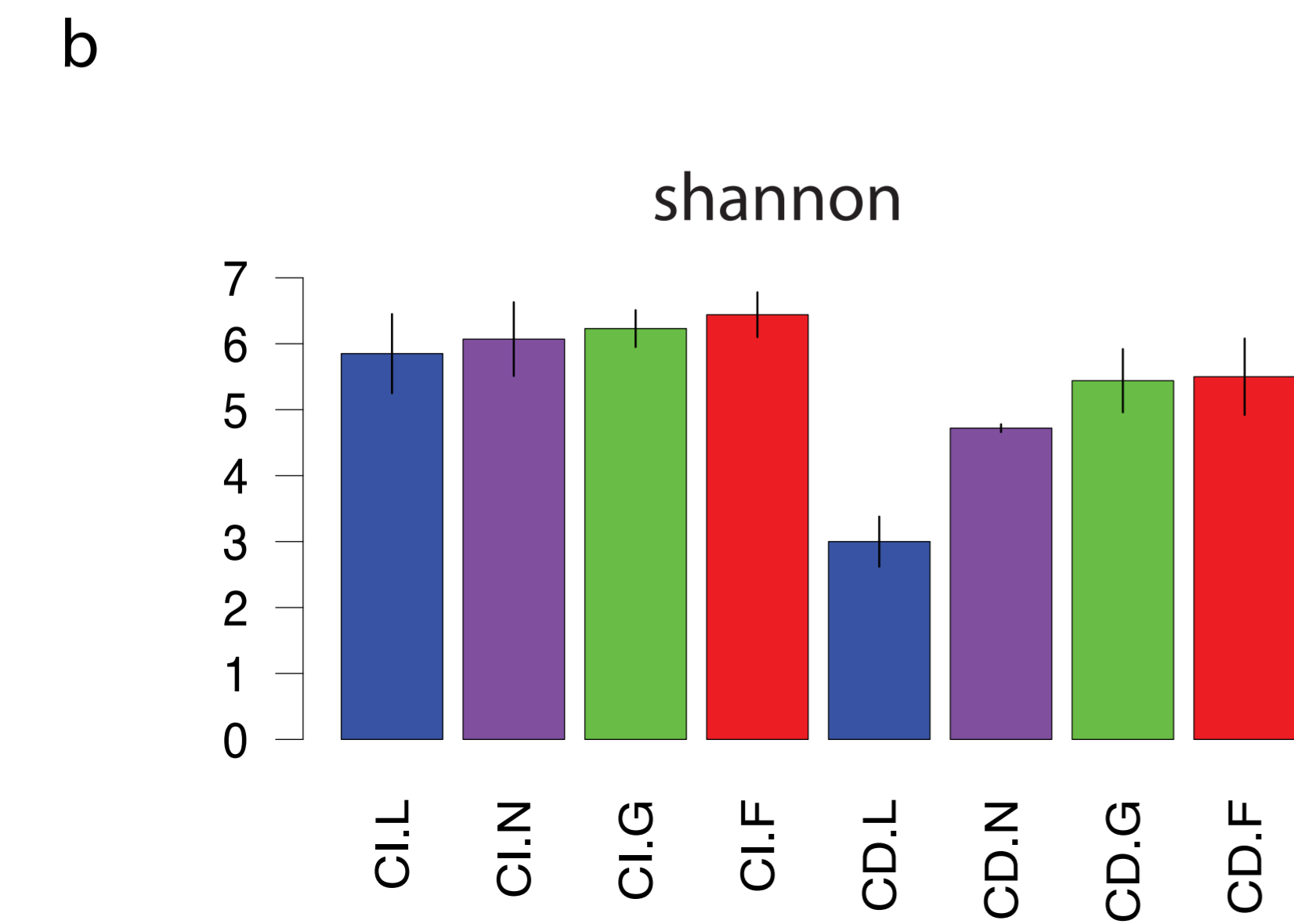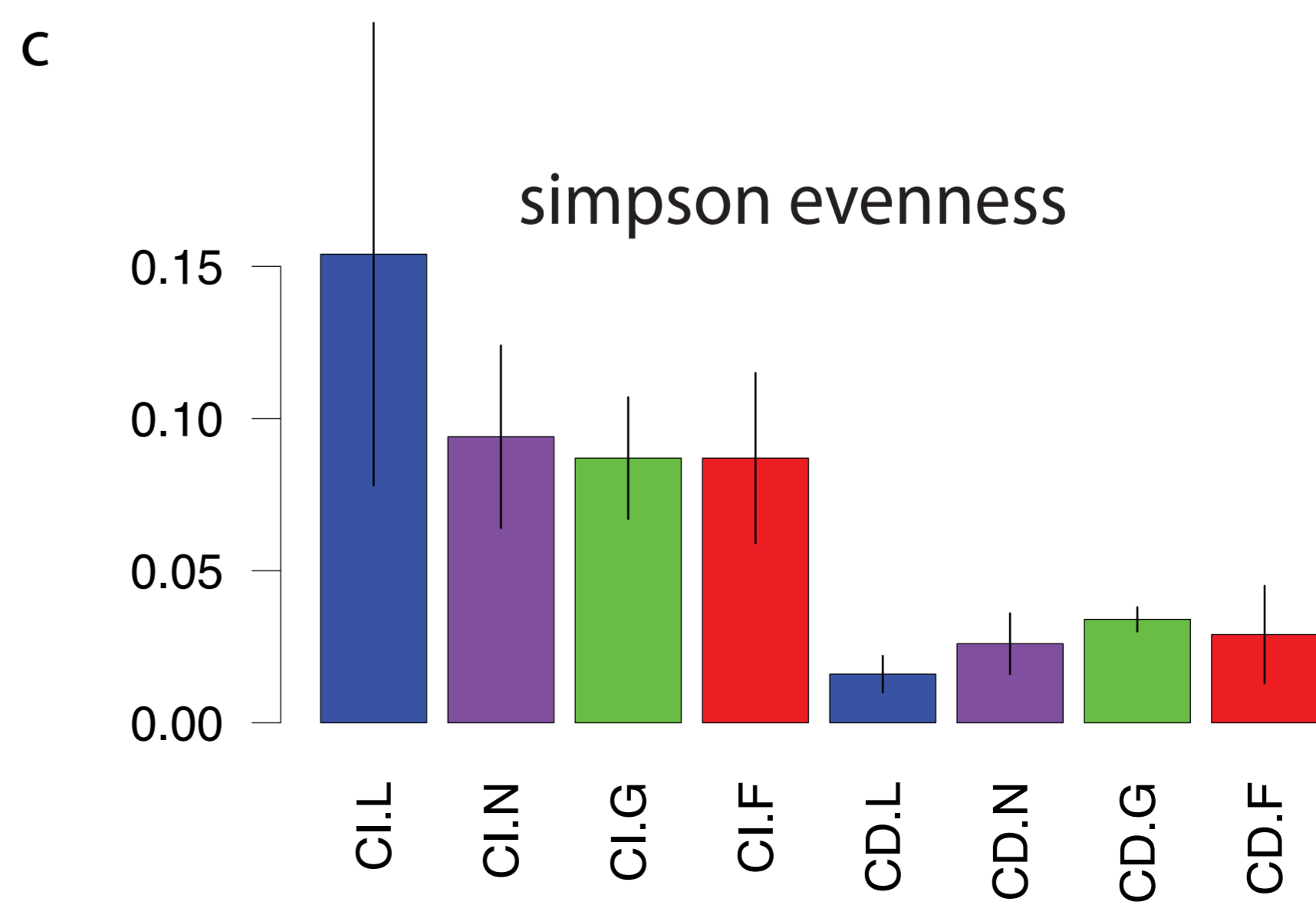

Supplement: FIG S1 [file msystems.00477-21-sf001.pdf]

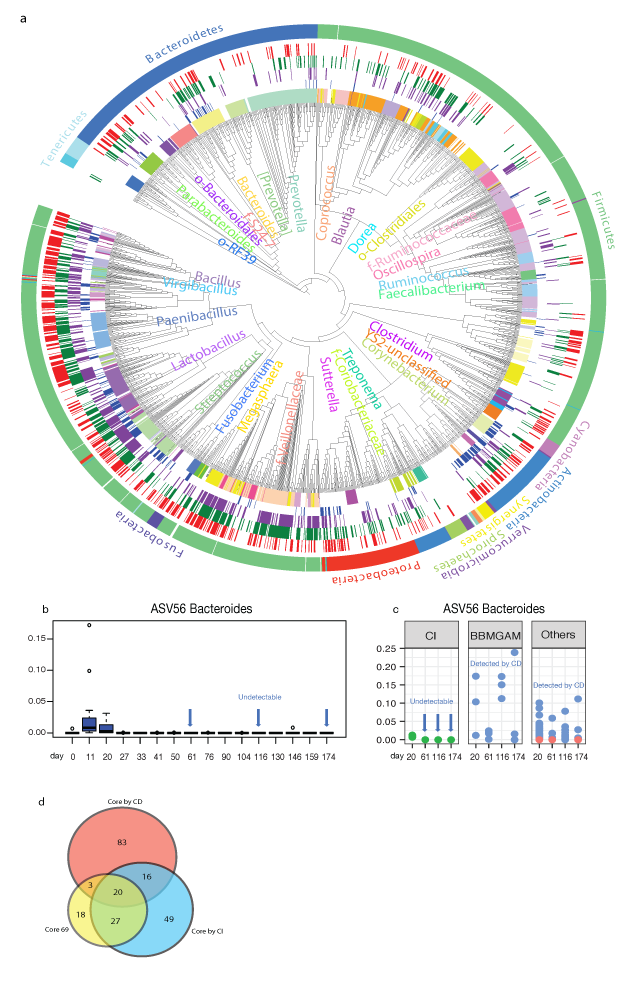

Supplement: FIG S2 [file msystems.00477-21-sf002.tif]

a

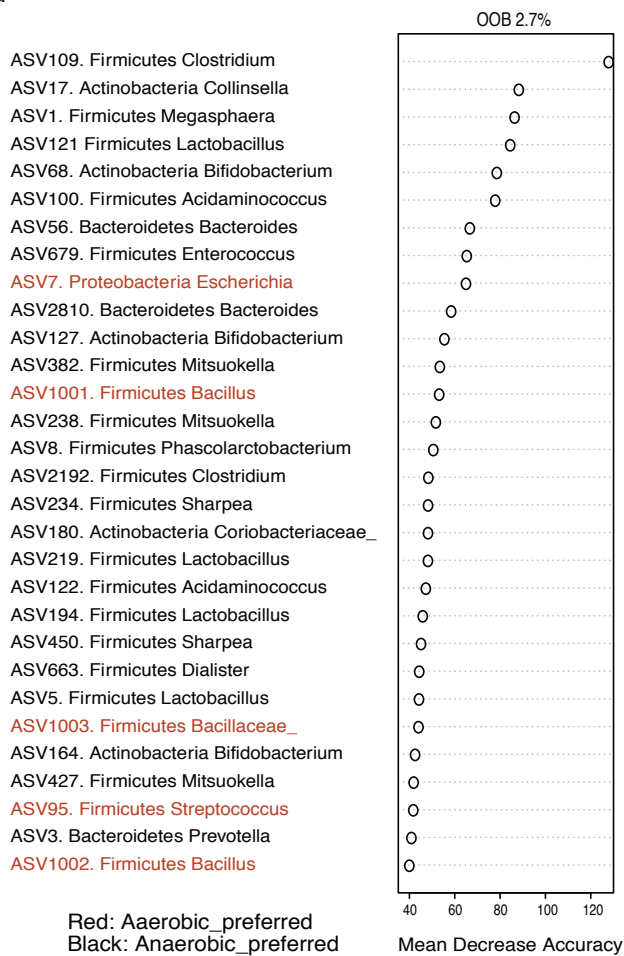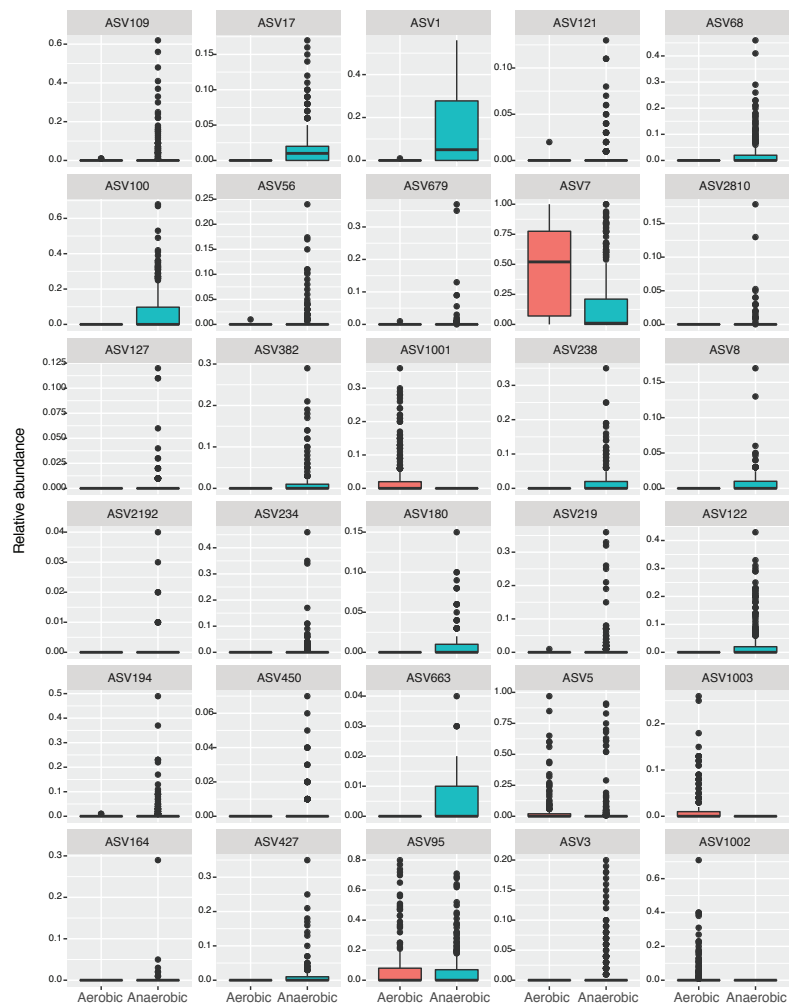

b

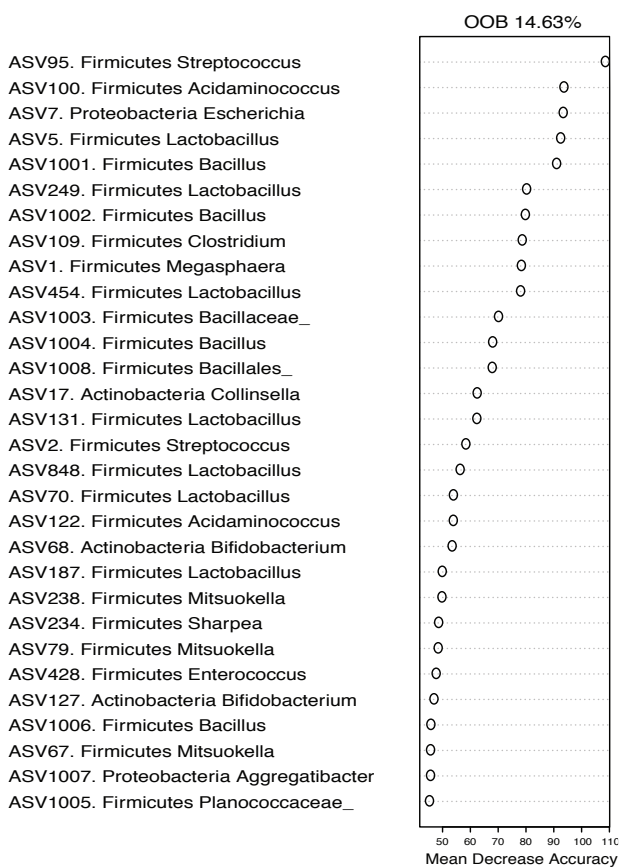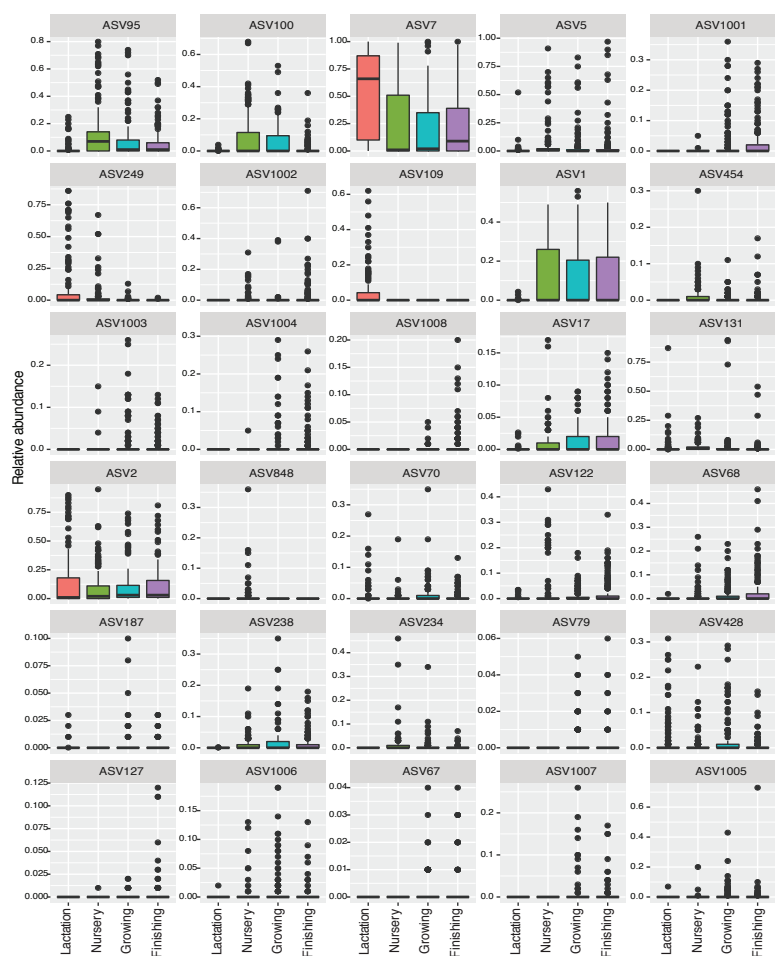

Supplement: FIG S3 [file msystems.00477-21-sf003.pdf]

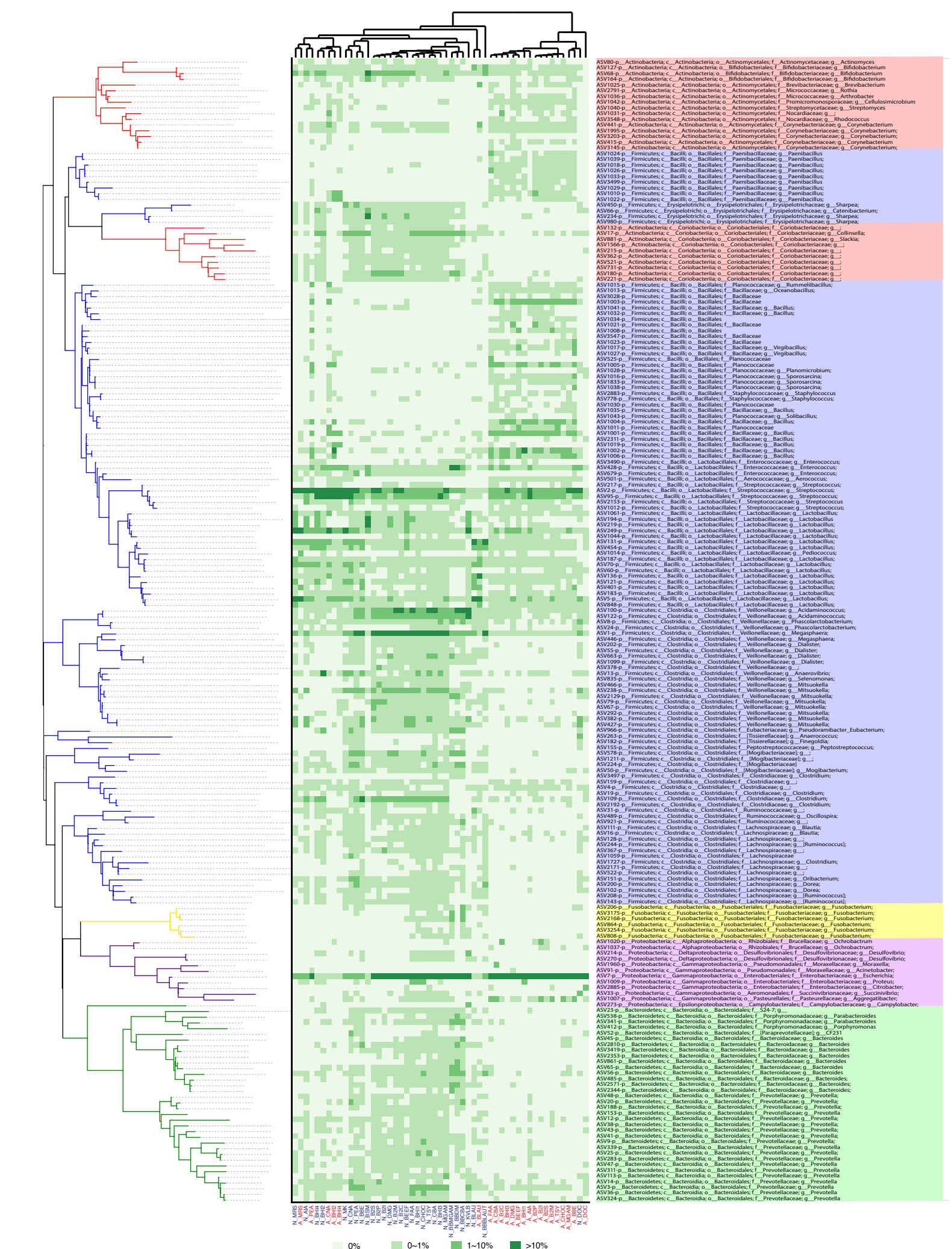

Supplement: FIG S4 [file msystems.00477-21-sf004.pdf]

Bray-Curtis

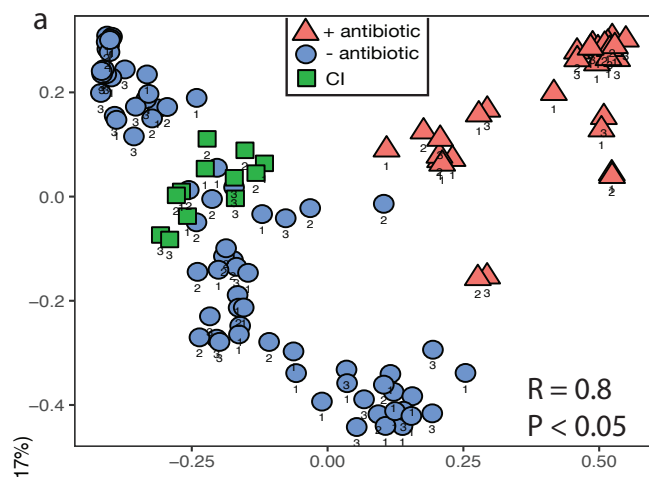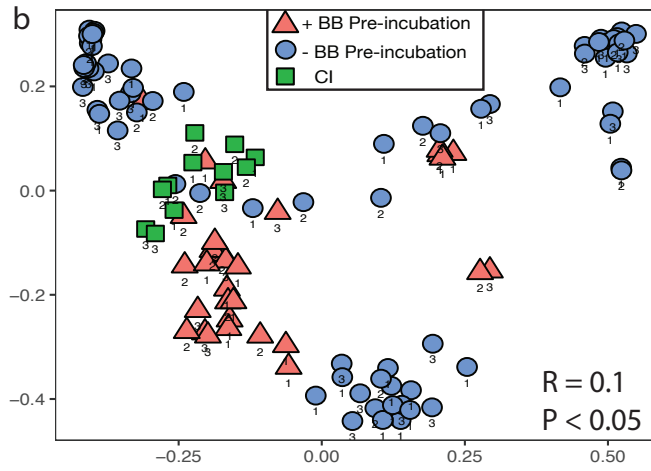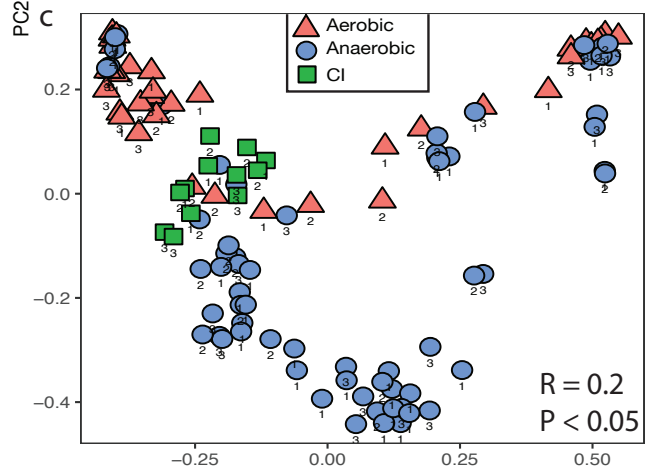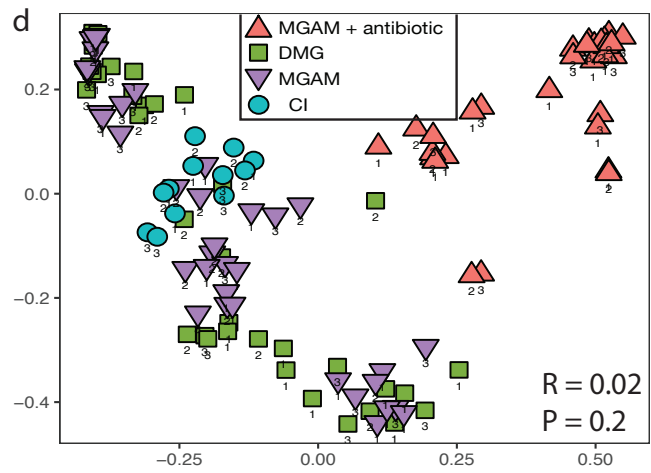

e

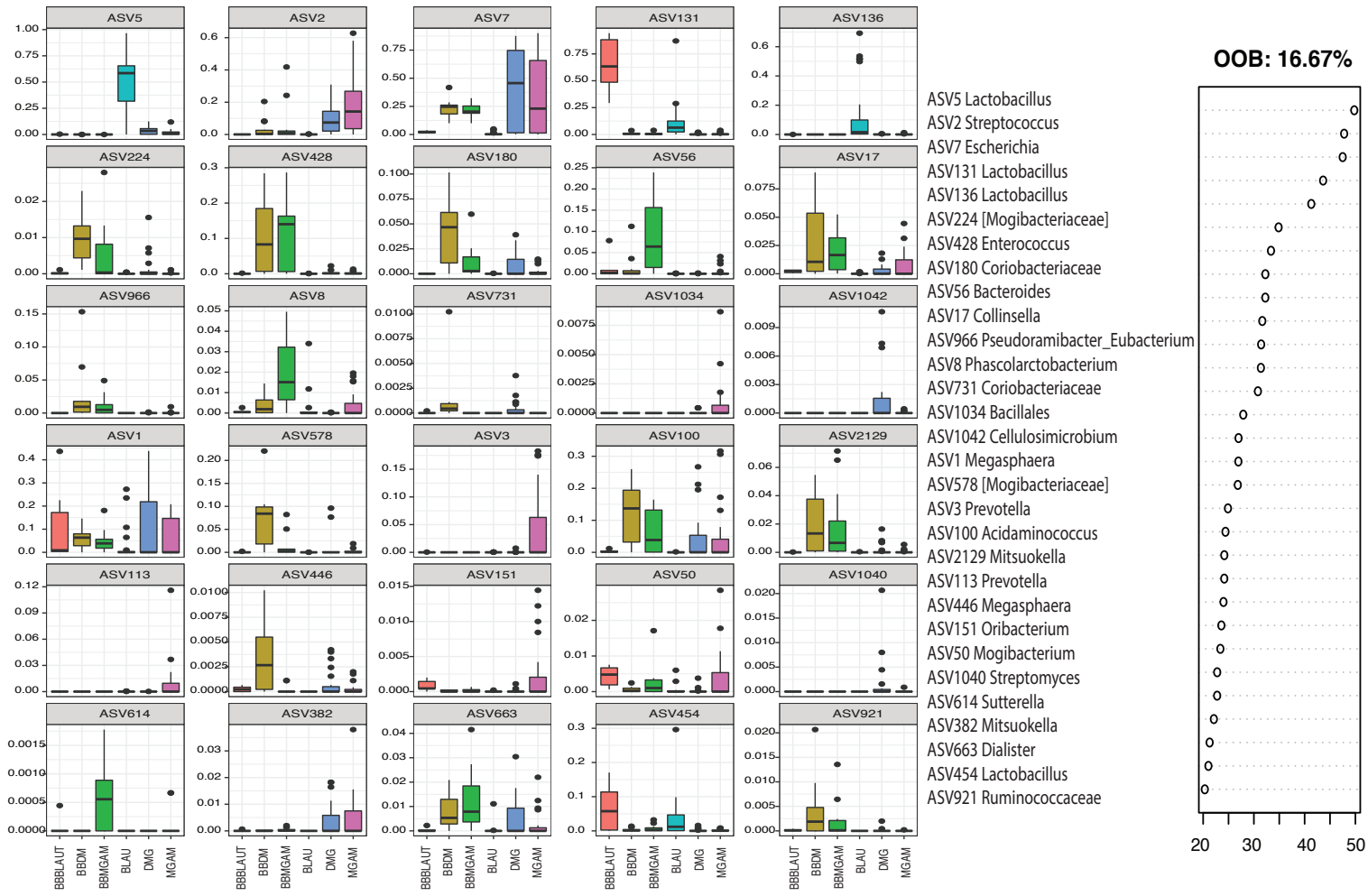

Supplement: FIG S5 [file msystems.00477-21-sf005.pdf]
