# Supplementary material for: Comprehensive Cultivation of the Swine Gut Microbiome Reveals High Bacterial Diversity and Guides Bacterial Isolation in Pigs
Source: mSystems. 2021 Jul 20;6(4):e00477-21. doi: 10.1128/mSystems.00477-21 (PMC8407297; doi:10.1128/mSystems.00477-21)
Supplement: TABLE S2 [file msystems.00477-21-st002.docx]

Supplemental Table 2. The best culture conditions and feces origin for the top 100 ASVs (unclassified taxa were removed) in the swine gut microbiome detected by culture-dependent methods.

| ID Familiy-Genus | Medium | Condition | Stage | Abundance |
| --- | --- | --- | --- | --- |
| ASV7 Enterobacteriaceae-Escherichia | DOC | Anaerobic (N) | Lactation | 0.9996 |
| ASV5 Lactobacillaceae-Lactobacillus | BLAU | Aerobic (A) | Finishing | 0.9658 |
| ASV2 Streptococcaceae-Streptococcus | BHI4 | A | Nursery | 0.9489 |
| ASV95 Streptococcaceae-Streptococcus | BHI2 | A | Nursery | 0.8044 |
| ASV100 Veillonellaceae-Acidaminococcus | KVLB | N | Nursery | 0.6773 |
| ASV1 Veillonellaceae-Megasphaera | BEEF | N | Growing | 0.5593 |
| ASV13 Veillonellaceae-Anaerovibrio | BHI2 | N | Growing | 0.5038 |
| ASV68 Bifidobacteriaceae-Bifidobacterium | BSM | N | Finishing | 0.4642 |
| ASV122 Veillonellaceae-Acidaminococcus | KVLB | N | Nursery | 0.4318 |
| ASV70 Lactobacillaceae-Lactobacillus | MRS | N | Growing | 0.3496 |
| ASV56 Bacteroidaceae-Bacteroides | BBMGAM | N | Finishing | 0.2389 |
| ASV3 Prevotellaceae-Prevotella | TSY | N | Growing | 0.2029 |
| ASV66 Erysipelotrichaceae-Catenibacterium | BSM | N | Finishing | 0.2009 |
| ASV45 Bacteroidaceae-Bacteroides | BBCBA | N | Lactation | 0.1967 |
| ASV33 Succinivibrionaceae-Succinivibrio | DOC | N | Nursery | 0.1847 |
| ASV17 Coriobacteriaceae-Collinsella | MK | N | Nursery | 0.1704 |
| ASV8 Veillonellaceae-Phascolarctobacterium | DOC | N | Growing | 0.1669 |
| ASV121 Lactobacillaceae-Lactobacillus | BHI4 | N | Growing | 0.1327 |
| ASV113 Prevotellaceae-Prevotella | MGAM | N | Nursery | 0.116 |
| ASV31 Ruminococcaceae- | BLAU | N | Nursery | 0.1078 |
| ASV20 Prevotellaceae-Prevotella | CNA | N | Nursery | 0.1069 |
| ASV14 Prevotellaceae-Prevotella | B2P | N | Nursery | 0.0916 |
| ASV60 Lactobacillaceae-Lactobacillus | CNA | N | Lactation | 0.0898 |
| ASV25 Prevotellaceae-Prevotella | TSY | N | Growing | 0.0744 |
| ASV36 Prevotellaceae-Prevotella | CNA | N | Growing | 0.0678 |
| ASV19 Clostridiaceae-Clostridium | MGAM | N | Finishing | 0.0673 |
| ASV9 Prevotellaceae-Prevotella | CHOC | N | Growing | 0.0627 |
| ASV41 Prevotellaceae-Prevotella | BBDM | N | Growing | 0.0607 |
| ASV52 [Paraprevotellaceae]-CF231 | CNA | N | Growing | 0.0578 |
| ASV79 Veillonellaceae-Mitsuokella | B2I | N | Finishing | 0.0556 |
| ASV67 Veillonellaceae-Mitsuokella | B2S | N | Growing | 0.0436 |
| ASV48 Prevotellaceae-Prevotella | CBA | N | Growing | 0.0356 |
| ASV128 Lachnospiraceae- | BLAU | N | Nursery | 0.0304 |
| ASV50 [Mogibacteriaceae]-Mogibacterium | MGAM | N | Finishing | 0.0284 |
| ASV16 Lachnospiraceae-Blautia | B2S | A | Nursery | 0.0282 |
| ASV24 Veillonellaceae-Phascolarctobacterium | TSY | N | Finishing | 0.0251 |
| ASV111 Lachnospiraceae-Blautia | BLAU | N | Growing | 0.0231 |
| ASV38 Prevotellaceae-Prevotella | CHOC | N | Finishing | 0.0198 |
| ASV102 Lachnospiraceae-Dorea | BBBLAUT | N | Finishing | 0.0193 |
| ASV55 Veillonellaceae-Dialister | MGAM | N | Growing | 0.0193 |
| ASV73 Prevotellaceae-Prevotella | CBA | N | Growing | 0.0169 |
| ASV43 Prevotellaceae-Prevotella | CNA | N | Growing | 0.0167 |
| ASV21 Prevotellaceae-Prevotella | CHOC | N | Finishing | 0.0153 |
| ASV4 Clostridiaceae- | BLAU | A | Finishing | 0.0147 |
| ASV12 Prevotellaceae-Prevotella | PEA | N | Finishing | 0.014 |
| ASV47 Prevotellaceae-Prevotella | CHOC | N | Growing | 0.014 |
| ASV42 Veillonellaceae- | FAA | N | Growing | 0.0109 |
| ASV23 S24-7- | CHOC | N | Finishing | 0.0104 |
| ASV10 [Paraprevotellaceae]-YRC22 | BLAU | N | Finishing | 0.0093 |
| ASV75 Clostridiaceae- | CNA | N | Finishing | 0.0087 |
| ASV6 Prevotellaceae-Prevotella | MRS | A | Lactation | 0.0084 |
| ASV15 Methanobacteriaceae-Methanobrevibacter | BLAU | N | Finishing | 0.0082 |
| ASV49 Prevotellaceae-Prevotella | MRS | N | Finishing | 0.0069 |
| ASV63 Prevotellaceae-Prevotella | BBE | N | Finishing | 0.0069 |
| ASV117 Lachnospiraceae-Blautia | BHI2 | N | Growing | 0.0067 |
| ASV18 Clostridiaceae-SMB53 | BLAU | A | Finishing | 0.0067 |
| ASV96 [Paraprevotellaceae]- | B2S | N | Nursery | 0.0067 |
| ASV27 Clostridiaceae-Clostridium | BLAU | A | Finishing | 0.0051 |
| ASV26 Turicibacteraceae-Turicibacter | BLAU | A | Finishing | 0.0042 |
| ASV29 Prevotellaceae-Prevotella | CHOC | N | Growing | 0.004 |
| ASV72 Prevotellaceae-Prevotella | CHOC | N | Finishing | 0.0036 |
| ASV99 Succinivibrionaceae-Succinivibrio | CHOC | N | Finishing | 0.0033 |
| ASV11 Planococcaceae-Rummeliibacillus | B2I | A | Finishing | 0.0031 |
| ASV22 Peptostreptococcaceae- | BLAU | A | Finishing | 0.0029 |
| ASV28 Ruminococcaceae-Oscillospira | BLAU | N | Lactation | 0.0027 |
| ASV37 Ruminococcaceae-Faecalibacterium | B2P | N | Nursery | 0.0024 |
| ASV51 Lachnospiraceae- | BLAU | A | Finishing | 0.0024 |
| ASV32 Succinivibrionaceae- | BHI2 | N | Finishing | 0.0018 |
| ASV46 [Paraprevotellaceae]-[Prevotella] | CNA | N | Growing | 0.0018 |
| ASV76 Methanobacteriaceae-Methanobrevibacter | BLAU | A | Lactation | 0.0018 |
| ASV101 Ruminococcaceae-Oscillospira | BBMGAM | N | Growing | 0.0013 |
| ASV69 [Paraprevotellaceae]-[Prevotella] | BBE | N | Finishing | 0.0013 |
| ASV34 p-2534-18B5- | BLAU | A | Lactation | 0.0011 |
| ASV40 Ruminococcaceae-Faecalibacterium prausnitzii | BLAU | A | Nursery | 0.0011 |
| ASV83 [Paraprevotellaceae]-[Prevotella] | BBE | N | Finishing | 0.0011 |
| ASV53 Christensenellaceae- | BLAU | A | Nursery | 0.0009 |
| ASV64 Methanobacteriaceae-Methanosphaera | DOC | N | Growing | 0.0009 |
| ASV114 Lachnospiraceae-Roseburia | AIA | A | Nursery | 0.0007 |
| ASV44 Methanobacteriaceae-Methanobrevibacter | BHI4 | A | Finishing | 0.0007 |
| ASV57 Bacteroidaceae-Bacteroides | BLAU | A | Lactation | 0.0007 |
| ASV86 Ruminococcaceae-Oscillospira | PEA | N | Finishing | 0.0007 |
| ASV103 Spirochaetaceae-Treponema | CBA | N | Finishing | 0.0004 |
| ASV104 Ruminococcaceae- | CHOC | N | Growing | 0.0004 |
| ASV118 Ruminococcaceae-Faecalibacterium | B2C | N | Growing | 0.0004 |
| ASV35 Spirochaetaceae-Treponema | CHOC | N | Growing | 0.0004 |
| ASV74 Veillonellaceae- | BLAU | N | Finishing | 0.0004 |
| ASV81 [Paraprevotellaceae]-YRC22 | BLAU | N | Finishing | 0.0004 |
| ASV39 Veillonellaceae- | B2M | A | Growing | 0.0002 |
| ASV77 Campylobacteraceae-Campylobacter | KVLB | N | Lactation | 0.0002 |
| ASV87 Ruminococcaceae- | BHI4 | A | Finishing | 0.0002 |
| ASV92 Ruminococcaceae-Oscillospira | BHI4 | N | Nursery | 0.0002 |
