# Supplementary material for: Comprehensive Cultivation of the Swine Gut Microbiome Reveals High Bacterial Diversity and Guides Bacterial Isolation in Pigs
Source: mSystems. 2021 Jul 20;6(4):e00477-21. doi: 10.1128/mSystems.00477-21 (PMC8407297; doi:10.1128/mSystems.00477-21)
Supplement: TABLE S3 [file msystems.00477-21-st003.docx]

Supplemental Table 3. PERMANOVA analysis of factors such as medium, oxygen, and donor pig age affecting swine culturomics.

|  | Df | SumsOfSqs | MeanSqs | F.Model | R2 | Pr(>F) |  |
| --- | --- | --- | --- | --- | --- | --- | --- |
| Medium | 29 | 57.29 | 1.98 | 10.62 | 0.27 | 0.001 | *** |
| Oxygen | 1 | 26.56 | 26.56 | 142.83 | 0.12 | 0.001 | *** |
| Pig age | 3 | 19.80 | 6.60 | 35.49 | 0.09 | 0.001 | *** |
| Residuals | 595 | 110.66 | 0.19 | 0.52 |  |  |  |
| Total | 628 | 214.31 | 1.00 |  |  |  |  |
|  | Df | SumsOfSqs | MeanSqs | F.Model | R2 | Pr(>F) |  |
| Oxygen | 1 | 26.36 | 26.36 | 141.73 | 0.12 | 0.001 | *** |
| Pig age | 3 | 19.71 | 6.57 | 35.33 | 0.09 | 0.001 | *** |
| Medium | 29 | 57.58 | 1.99 | 10.68 | 0.27 | 0.001 | *** |
| Residuals | 595 | 110.66 | 0.19 | 0.52 |  |  |  |
| Total | 628 | 214.31 | 1 |  |  |  |  |
|  | Df | SumsOfSqs | MeanSqs | F.Model | R2 | Pr(>F) |  |
| Pig age | 3 | 19.87 | 6.62 | 35.62 | 0.09 | 0.001 | *** |
| Medium | 29 | 57.23 | 1.97 | 10.61 | 0.27 | 0.001 | *** |
| Oxygen | 1 | 26.54 | 26.54 | 142.72 | 0.12 | 0.001 | *** |
| Residuals | 595 | 110.66 | 0.19 | 0.52 |  |  |  |
| Total | 628 | 214.31 | 1 |  |  |  |  |
|  | Df | SumsOfSqs | MeanSqs | F.Model | R2 | Pr(>F) |  |
| Medium | 29 | 57.29 | 1.98 | 10.62 | 0.27 | 0.001 | *** |
| Pig age | 3 | 19.82 | 6.61 | 35.53 | 0.09 | 0.001 | *** |
| Oxygen | 1 | 26.54 | 26.54 | 142.72 | 0.12 | 0.001 | *** |
| Residuals | 595 | 110.66 | 0.19 | 0.52 |  |  |  |
| Total | 628 | 214.31 | 1 |  |  |  |  |
|  | Df | SumsOfSqs | MeanSqs | F.Model | R2 | Pr(>F) |  |
| Oxygen | 1 | 26.36 | 26.36 | 141.73 | 0.12 | 0.001 | *** |
| Medium | 29 | 57.49 | 1.98 | 10.66 | 0.27 | 0.001 | *** |
| Pig age | 3 | 19.80 | 6.60 | 35.49 | 0.09 | 0.001 | *** |
| Residuals | 595 | 110.66 | 0.19 | 0.52 |  |  |  |
| Total | 628 | 214.31 | 1 |  |  |  |  |
|  | Df | SumsOfSqs | MeanSqs | F.Model | R2 | Pr(>F) |  |
| Pig age | 3 | 19.87 | 6.62 | 35.62 | 0.09 | 0.001 | *** |
| Oxygen | 1 | 26.20 | 26.20 | 140.87 | 0.12 | 0.001 | *** |
| Medium | 29 | 57.58 | 1.99 | 10.68 | 0.27 | 0.001 | *** |
| Residuals | 595 | 110.66 | 0.19 | 0.52 |  |  |  |
| Total | 628 | 214.31 | 1 |  |  |  |  |
|  |  |  |  |  |  |  |  |
|  | F.Model_mean_ | R2_mean_ | Pr(>F)_mean_ |  |  |  |  |
| Medium | 10.64 | 0.27 | 0.001 |  |  |  |  |
| Oxygen | 142.1 | 0.12 | 0.001 |  |  |  |  |
| Pig age | 35.51 | 0.09 | 0.001 |  |  |  |  |
