# Supplementary material for: Comprehensive Cultivation of the Swine Gut Microbiome Reveals High Bacterial Diversity and Guides Bacterial Isolation in Pigs
Source: mSystems. 2021 Jul 20;6(4):e00477-21. doi: 10.1128/mSystems.00477-21 (PMC8407297; doi:10.1128/mSystems.00477-21)
Supplement: TABLE S4 [file msystems.00477-21-st004.docx]

Supplemental Table 4. Recovery rates, i.e., proportions of cultivable ASVs in the culture-independent group, are shown at each time point, with two thresholds set at 0.001 and 0.01 for low abundant microbes (a). Percentages of bacterial families under anaerobic and aerobic conditions at four time points (b).

a.

|  | Lactation | Nursery | Growing | Finishing | Average |
| --- | --- | --- | --- | --- | --- |
| Recovery rate, rb > 0.001 | 70% | 100% | 93% | 87% | 87% |
| Recovery rate, rb > 0.01 | 96% | 100% | 100% | 100% | 99% |

b.

| Family | Lactation Aerobic | Nursery Aerobic | Growing Aerobic | Finishing Aerobic | Lactation Anaerobic | Nursery Anaerobic | Growing Anaerobic | Finishing Anaerobic |
| --- | --- | --- | --- | --- | --- | --- | --- | --- |
| Enterobacteriaceae | 64.4% | 47.2% | 36.3% | 42.6% | 46.0% | 4.9% | 4.7% | 7.9% |
| Streptococcaceae | 14.0% | 27.9% | 22.1% | 13.1% | 14.8% | 15.3% | 13.8% | 18.8% |
| Bacillaceae | 0.1% | 3.9% | 17.7% | 22.0% | 0.2% | 0.0% | 0.1% | 0.0% |
| Veillonellaceae | 0.1% | 0.2% | 0.0% | 0.0% | 0.6% | 47.9% | 41.7% | 32.3% |
| Coriobacteriaceae | 0.0% | 0.0% | 0.1% | 0.1% | 0.1% | 2.7% | 3.3% | 5.3% |
| Bifidobacteriaceae | 0.0% | 0.0% | 0.0% | 0.0% | 0.5% | 1.7% | 2.8% | 6.0% |
| Bacteroidaceae | 0.1% | 0.2% | 0.0% | 0.0% | 4.3% | 0.4% | 1.0% | 1.0% |
| Erysipelotrichaceae | 0.0% | 0.0% | 0.0% | 0.1% | 0.1% | 2.4% | 2.3% | 0.9% |
