# Supplementary material for: Comprehensive Cultivation of the Swine Gut Microbiome Reveals High Bacterial Diversity and Guides Bacterial Isolation in Pigs
Source: mSystems. 2021 Jul 20;6(4):e00477-21. doi: 10.1128/mSystems.00477-21 (PMC8407297; doi:10.1128/mSystems.00477-21)
Supplement: TABLE S5 [file msystems.00477-21-st005.docx]

Supplemental Table 5. Anaerobic medium BEEF recovered similar culturable bacterial profiles using both humans (16) and pigs’ samples.

|  | BEEF_anaerobic condition | |
| --- | --- | --- |
|  | Human* | swine |
| *Bifidobacteriaceae* | >10% | 6% |
| *Coriobacteriaceae* | >1% | 3% |
| *Streptococcaceae* | >1% | 9% |
| *Clostridiaceae* | >1% | 4% |
| *Veillonellaceae* | >1% | 41% |
| *Erysipelotrichaceae* | >10% | 2% |
| *Enterobacteriaceae* | >10% | 20% |

Reference:

16. Lau JT, Whelan FJ, Herath I, Lee CH, Collins SM, Bercik P, et al. Capturing the diversity of the human gut microbiota through culture-enriched molecular profiling. Genome Medicine. 2016;8(1):72.
