# Supplementary material for: Comprehensive Cultivation of the Swine Gut Microbiome Reveals High Bacterial Diversity and Guides Bacterial Isolation in Pigs
Source: mSystems. 2021 Jul 20;6(4):e00477-21. doi: 10.1128/mSystems.00477-21 (PMC8407297; doi:10.1128/mSystems.00477-21)
Supplement: TABLE S1 [file msystems.00477-21-st001.docx]

Supplemental Table 1. Nutritional composition was formulated based on NRC (2012) nutrient requirements. Diets were formulated based on growth status, including three nursery phases (Np1, Np2, and Np3), two growing phases (Gp1 and Gp2), and two finishing phases (Fp1 and Fp2).

|  | Np1 | Np2 | Np3 | Gp1 | Gp2 | Fp1 | Fp2 |
| --- | --- | --- | --- | --- | --- | --- | --- |
| CP (%) | 22.91 | 25.37 | 23.64 | 19.56 | 16.70 | 15.08 | 14.09 |
| Crude fat(%) | 5.39 | 6.60 | 6.47 | 3.80 | 3.94 | 4.03 | 4.08 |
| Iodine value | 82.32 | 85.44 | 85.68 | 87.55 | 88.00 | 88.48 | 88.68 |
| Crude fiber(%) | 1.81 | 3.47 | 3.51 | 3.37 | 3.23 | 3.16 | 3.11 |
| NDF(%) | 5.59 | 11.22 | 11.49 | 11.90 | 11.96 | 12.02 | 12.05 |
| ADF(%) | 2.44 | 4.75 | 4.84 | 4.68 | 4.51 | 4.42 | 4.36 |
| Total Arginine (%) | 1.35 | 1.55 | 1.45 | 1.13 | 0.91 | 0.79 | 0.71 |
| Total Cys (%) | 0.39 | 0.40 | 0.38 | 0.33 | 0.29 | 0.27 | 0.26 |
| Total Histidine (%) | 0.61 | 0.68 | 0.63 | 0.52 | 0.45 | 0.41 | 0.38 |
| Total Isoleucine (%) | 0.97 | 1.04 | 0.97 | 0.78 | 0.64 | 0.57 | 0.52 |
| Total Leucine (%) | 1.92 | 2.21 | 2.09 | 1.83 | 1.64 | 1.54 | 1.47 |
| Total Lysine (%) | 1.60 | 1.62 | 1.47 | 1.18 | 0.97 | 0.83 | 0.77 |
| Total M+C (%) | 0.94 | 0.96 | 0.87 | 0.67 | 0.59 | 0.55 | 0.53 |
| Total Met (%) | 0.55 | 0.56 | 0.49 | 0.35 | 0.30 | 0.28 | 0.27 |
| Total P (%) | 0.68 | 0.58 | 0.49 | 0.45 | 0.41 | 0.38 | 0.37 |
| Total Phenylalanine (%) | 1.06 | 1.22 | 1.15 | 0.94 | 0.80 | 0.72 | 0.67 |
| Total Threonine (%) | 1.02 | 1.03 | 0.93 | 0.76 | 0.64 | 0.56 | 0.54 |
| Total Tryptophan (%) | 0.30 | 0.29 | 0.27 | 0.21 | 0.17 | 0.15 | 0.14 |
| Total Tyrosine (%) | 0.74 | 0.87 | 0.81 | 0.67 | 0.58 | 0.53 | 0.49 |
| Total Valine (%) | 1.10 | 1.18 | 1.09 | 0.91 | 0.77 | 0.70 | 0.65 |
| Available P (%) | 0.46 | 0.31 | 0.23 | 0.20 | 0.19 | 0.17 | 0.16 |
| Aval. P (%) with phytase | 0.55 | 0.41 | 0.32 | 0.30 | 0.28 | 0.26 | 0.25 |
| Ca (%) | 0.85 | 0.75 | 0.65 | 0.61 | 0.59 | 0.54 | 0.50 |
| Na (%) | 0.40 | 0.30 | 0.28 | 0.21 | 0.21 | 0.21 | 0.20 |
| K(%) | 1.11 | 1.00 | 0.99 | 0.80 | 0.66 | 0.58 | 0.53 |
| CL(%) | 0.63 | 0.52 | 0.50 | 0.37 | 0.34 | 0.32 | 0.31 |
| Sulfur(%) | 0.30 | 0.22 | 0.20 | 0.16 | 0.13 | 0.11 | 0.10 |
| Zinc(ppm) | 194.17 | 200.11 | 196.77 | 193.81 | 191.47 | 135.08 | 134.20 |
| Manganese(ppm) | 54.41 | 58.55 | 57.89 | 54.67 | 52.45 | 37.86 | 36.88 |
| Copper(ppm) | 23.93 | 24.07 | 23.81 | 22.65 | 21.81 | 15.85 | 15.55 |
| Iodine(ppm) | 0.30 | 0.30 | 0.30 | 0.30 | 0.30 | 0.20 | 0.20 |
| Selenium(ppm) | 0.56 | 0.54 | 0.48 | 0.46 | 0.44 | 0.34 | 0.33 |
| Lactose | 17.90 | 0.00 | 0.00 | 0.00 | 0.00 | 0.00 | 0.00 |
| Ca/P | 1.25 | 1.31 | 1.33 | 1.35 | 1.42 | 1.41 | 1.36 |
| Ca/AP | 1.85 | 2.41 | 2.89 | 2.97 | 3.16 | 3.26 | 3.20 |
| Ca/aP with phytase | 1.54 | 1.85 | 2.04 | 2.03 | 2.10 | 2.08 | 2.00 |
| NSNG NE (Mcal/lb) | 1.18 | 1.14 | 1.14 | 1.11 | 1.13 | 1.14 | 1.15 |

Note: CP, crude protein; NDF, neutral detergent fiber; ADF, acid detergent fiber; NE, net energy.
